# A dual catalytic strategy for carbon–phosphorus cross-coupling *via* gold and photoredox catalysis†
†Electronic supplementary information (ESI) available: Materials, full experimental details and characterisation. See DOI: 10.1039/c4sc03092c


**DOI:** 10.1039/c4sc03092c

**Published:** 2014-11-25

**Authors:** Ying He, Hongmiao Wu, F. Dean Toste

**Affiliations:** a Institute of Chemistry and BioMedical Sciences , Nanjing University , Nanjing , 210046 , China; b Department of Chemistry , University of California , Berkeley , CA 94720 , USA . Email: fdtoste@berkeley.edu

## Abstract

A new method for the *P*-arylation of aryldiazonium salts with *H*-phosphonates *via* dual gold and photoredox catalysis is described. The reaction proceeds smoothly at room temperature in the absence of base and/or additives, and offers an efficient approach to arylphosphonates. The reaction is proposed to proceed through a photoredox-promoted generation of an electrophilic arylgold(iii) intermediate that undergoes coupling with the *H*-phosphonate nucleophile.

## Abstract 2

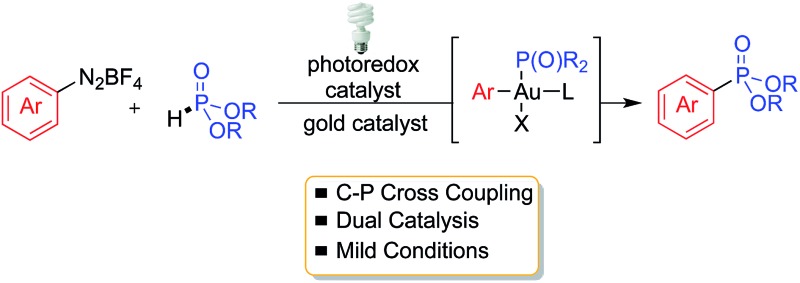
A new method for the *P*-arylation of aryldiazonium salts with *H*-phosphonates *via* dual gold and photoredox catalysis is described.

## Introduction

During the past decade, homogeneous gold reactions based on Au(i) or Au(iii) catalysis have emerged as an extraordinary tool to create molecular complexity. In these reactions, gold most-commonly acts as a redox-neutral and carbophilic π-acid that activates carbon–carbon multiple bonds towards nucleophilic attack.^1^ Alternatively, gold-catalyzed transformations employing a stoichiometric external oxidant, such as Selectfluor, have allowed entry into pathways involving Au(i)/Au(iii);^2^ however, the majority of these reactions still involve intermediates generated from activation of a carbon–carbon π-bond. The requirement for stoichiometric amounts of strong oxidizing reagents has generally limited the chemistry to π-bonds and aromatic compounds.^3^ Recently, stepwise oxidation of gold(i) complexes by photoredox-generated^4^ radical species has emerged as an alternative strategy for accessing Au(i)/Au(iii) coupling reactions.^5^

Organophosphorus compounds have drawn increasing attention due to their broad applications in biological, pharmaceutical, and material sciences.^6^ These compounds are commonly accessed through transition metal-catalyzed coupling processes.^7^ More recently, the desired coupling has been achieved through the reaction of phosphonate esters^8^ or phosphine oxides^9^ with highly electrophilic arylcopper(iii) intermediates^10^ generated from oxidation of copper(i) with diaryliodionium(iii) salts [eqn (1)]. While the reported combined photoredox/gold-catalyzed reactions have relied on the intervention of carbon–carbon π-bonds [eqn (2)], we hypothesized that the gold(iii) intermediates generated in this manner might also engage in coupling reactions with other nucleophilic species [eqn (3)].^11^
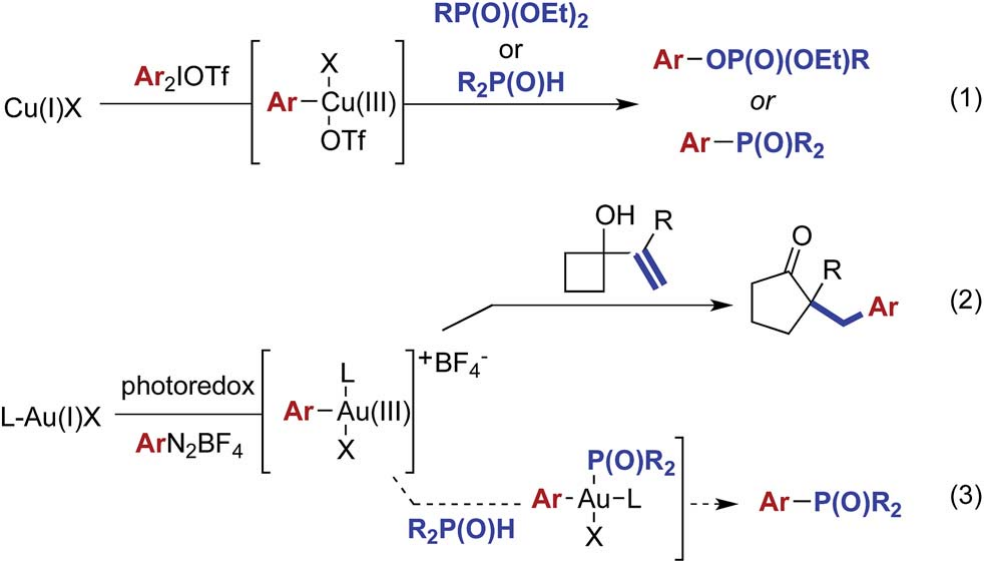


## Results and discussions

To this end, we explored the dual photoredox/gold-catalyzed coupling reaction of *p*-tolyldiazonium^12^ with diethyl phosphite (Table 1). The initial screening of solvents found that the desired product was formed in 37% yield when acetonitrile was employed as solvent (Table 1, entry 1). Other solvents commonly employed in photocatalysis, such as DMF and EtOH, were also tested and afforded the product in 50% and 65%, respectively (Table 1, entries 2 and 3). In order to exploit the better solubility of diazonium salts and tautomerization of *H*-phosphonates^13^ in polar solvents, we explored whether a solvent mixture with ethanol might improve the reaction outcome. The co-solvent consisting of MeCN/EtOH (4 : 1) gave the best result, affording the arylphosphonate in 82% yield (Table 1, entries 4–7). Lower yields were obtained when Ru(bpy)_3_Cl_2_ or Ir(ppy)_3_ were used as the photocatalyst (Table 1, entries 8 and 9). No product was observed with IPrAuCl as the gold catalyst (Table 1, entry 10), and reducing the amount of photocatalyst or gold catalyst both gave lower yields (Table 1, entries 11 and 12). Moreover, no product was detected when the reaction was performed in the absence of the gold catalyst (Table 1, entry 13) and significantly reduced yields of **3a** were observed in the absence of the Ru(bpy)_3_(PF_6_)_2_ and/or light (Table 1, entries 14 and 15). We also examined replacing the gold catalyst with those derived from palladium, silver or copper salts; however, only moderate yield was obtained when Pd(OAc)_2_ was used (Table 1, entry 16), and no product was detected with other catalysts even when the reaction time was prolonged to 16 h (Table 1, entries 17–22).

**Table 1:** Optimization of reaction conditions^*a*^

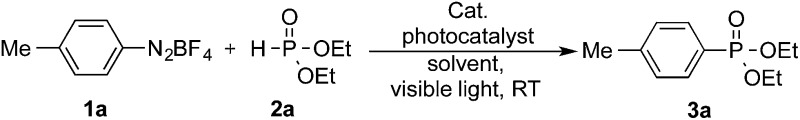					
Entry	Cat.	Photocatalyst	Solvent	Time (h)	Yield^*b*^ (%)
1	Ph_3_PAuCl	Ru(bpy)_3_(PF_6_)_2_	MeCN	4	37
2	Ph_3_PAuCl	Ru(bpy)_3_(PF_6_)_2_	DMF	4	50
3	Ph_3_PAuCl	Ru(bpy)_3_(PF_6_)_2_	EtOH	4	65
4	Ph_3_PAuCl	Ru(bpy)_3_(PF_6_)_2_	DMF : EtOH = 4 : 1	4	49
5	Ph_3_PAuCl	Ru(bpy)_3_(PF_6_)_2_	MeCN : EtOH = 4 : 1	4	82
6	Ph_3_PAuCl	Ru(bpy)_3_(PF_6_)_2_	MeCN : EtOH = 1 : 1	4	61
7	Ph_3_PAuCl	Ru(bpy)_3_(PF_6_)_2_	MeCN : EtOH = 9 : 1	4	58
8	Ph_3_PAuCl	Ru(bpy)_3_Cl_2_	MeCN : EtOH = 4 : 1	4	77
9	Ph_3_PAuCl	Ir(ppy)_3_	MeCN : EtOH = 4 : 1	4	24
10	IPrAuCl	Ru(bpy)_3_(PF_6_)_2_	MeCN : EtOH = 4 : 1	4	0
11^*c*^	Ph_3_PAuCl	Ru(bpy)_3_(PF_6_)_2_	MeCN : EtOH = 4 : 1	4	73
12^*d*^	Ph_3_PAuCl	Ru(bpy)_3_(PF_6_)_2_	MeCN : EtOH = 4 : 1	4	54
13	—	Ru(bpy)_3_(PF_6_)_2_	MeCN : EtOH = 4 : 1	4	0
14	Ph_3_PAuCl	—	MeCN : EtOH = 4 : 1	4	<10
15^*e*^	Ph_3_PAuCl	Ru(bpy)_3_(PF_6_)_2_	MeCN : EtOH = 4 : 1	4	<5
16	Pd(OAc)_2_	Ru(bpy)_3_(PF_6_)_2_	MeCN : EtOH = 4 : 1	4	43
17	AgNTf_2_	Ru(bpy)_3_(PF_6_)_2_	MeCN : EtOH = 4 : 1	16	0
18	AgBF_4_	Ru(bpy)_3_(PF_6_)_2_	MeCN : EtOH = 4 : 1	16	0
19^*f*^	AgBF_4_	Ru(bpy)_3_(PF_6_)_2_	MeCN : EtOH = 4 : 1	16	0
20	AgOTf	Ru(bpy)_3_(PF_6_)_2_	MeCN : EtOH = 4 : 1	16	0
21	Cu(OAc)_2_	Ru(bpy)_3_(PF_6_)_2_	MeCN : EtOH = 4 : 1	16	0
22	CuI	Ru(bpy)_3_(PF_6_)_2_	MeCN : EtOH = 4 : 1	16	0

^*a*^Reactions were carried out at room temperature with a 26 W household bulb, **1a** (0.3 mmol), **2a** (0.1 mmol), cat. (10 mol%), photocatalyst (2 mol%), degassed solvent (0.5 ml), N_2_ atmosphere, rt.

^*b*^Isolated yields.

^*c*^1 mol% Ru(bpy)_3_(PF_6_)_2_ was used.

^*d*^5 mol% Ph_3_PAuCl was used.

^*e*^Reaction run in the dark.

^*f*^10 mol% PPh_3_ was used as the ligand.

With the optimized conditions in hand, we investigated the scope of the diazonium substrates. Aryldiazoniums salts bearing electron-donating groups at their *para*-positions, such as methyl, phenyl and methoxy, were coupled with diethyl phosphite affording the corresponding products in good to excellent yields (Table 2, compounds **3a–3c**). However, the reactivity was dramatically decreased when aryldiazoniums salts containing strong withdrawing groups such as –CF_3_ and –NO_2_ were used (Table 2, compounds **3k** and **3l**). As expected, the *P*-arylation using aryl diazoniums with halogen in their *para* and *meta* positions proceeded efficiently, and yields of 71–86% were obtained (Table 2, compounds **3e–3g**). However, 2-bromophenyl diazonium was less reactive comparatively and 37% yield of the product was obtained (Table 2, compound **3h**). The naphthyl phosphonate was isolated in 72% yield under the standard reaction conditions (Table 2, compound **3i**).

**Table 2:** *P*-arylation of various aryldiazoium salts with diethyl phosphite^*a*^

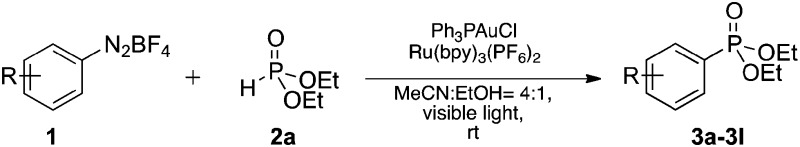
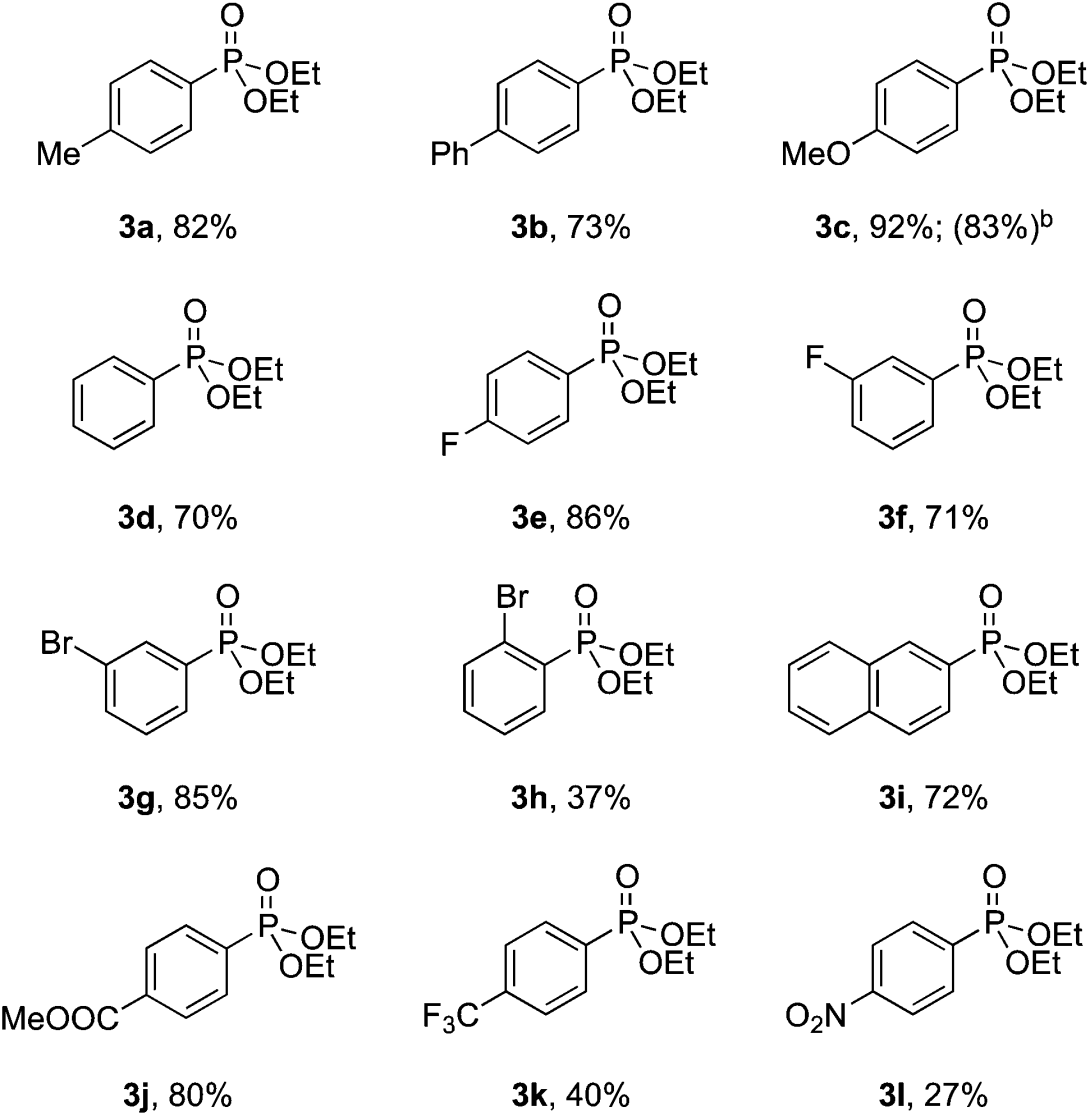

^*a*^Reaction conditions: 1 (0.3 mmol), **2a** (0.1 mmol), Ph_3_PAuCl (10 mol%), Ru(bpy)_3_(PF_6_)_2_ (2 mol%), degassed MeCN : EtOH = (4 : 1) (0.5 ml), N_2_ atmosphere, visible light, rt. for **4h**, isolated yields for all products.

^*b*^1 (9 mmol), **2a** (3 mmol), Ph_3_PAuCl (8 mol%), Ru(bpy)_3_(PF_6_)_2_ (2 mol%); isolated yields.

We next turned our attention to an evaluation of the scope and limitations of our reaction with different types of P(O)H compounds. As seen in Table 3, aryl diazonium salts with electron-donating, electron-withdrawing and halogen substituents reacted with *H*-phosphonate diesters bearing different alkyl groups efficiently, and yields of 74–90% were obtained (Table 3, entries 1–5). The reaction also proceeded smoothly with dibenzyl and ethyl phenylphosphinate as coupling partners (Table 3, entries 6 and 8). The more challenging coupling of *H*-phosphonate diphenylester also occurred under the gold-catalyzed reaction conditions, albeit it in diminished yield (Table 3, entry 7).

**Table 3:** Scope studies of various P(O)H compounds and aryldiazonium salts^*a*^

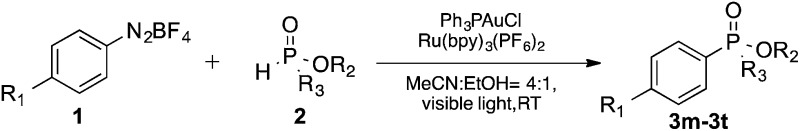			
Entry	R_1_	P(O)H compounds	Yield^*b*^ (%)
1^*c*^	OMe	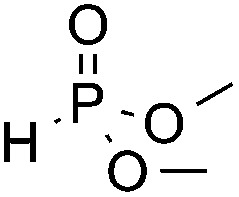	**3m**, 90
2^*c*^	F	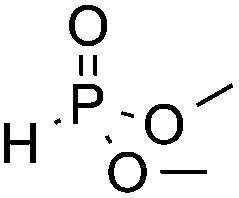	**3n**, 74
3^*c*^	COOMe	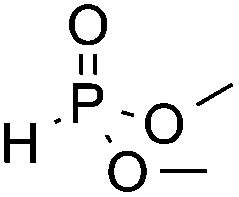	**3o**, 88
4^*d*^	OMe	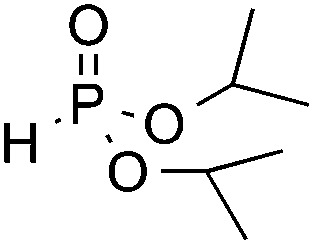	**3p**, 81
5^*d*^	F	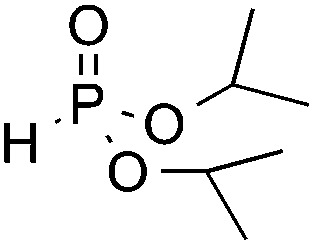	**3q**, 84
6	OMe	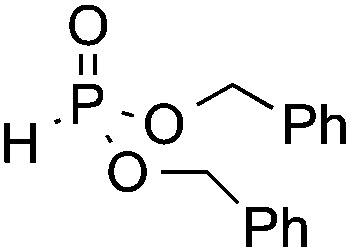	**3r**, 50
7	OMe	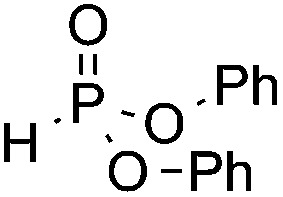	**3s**, 31
8	OMe	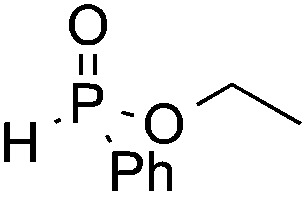	**3t**, 82

^*a*^Reaction conditions: 1 (0.3 mmol), 2 (0.1 mmol), Ph_3_PAuCl (10 mol%), Ru(bpy)_3_(PF_6_)_2_ (2 mol%), degassed MeCN : EtOH = (4 : 1) (0.5 ml), N_2_ atmosphere, visible light, rt. for **4h**.

^*b*^Isolated yield.

^*c*^MeCN : MeOH = (4 : 1) (0.5 ml) as the solvent.

^*d*^MeCN : ^i^PrOH = (4 : 1) (0.5 ml) as the solvent.

Interestingly, phenyl phosphinic acid was also a competent nucleophile for this reaction yielding products of a three-component coupling between the diazonium salt, arylphosphinic acid and the alkyl alcohol solvent [eqn (4)].

Additionally, the intermediate aryl diazonium salt can be generated without purification from the corresponding aniline. For example, **3e** was obtained in 69% through one-pot, two-step procedure for the diazotization and *P*-arylation of 4-fluoroaniline, compared with 86% under standard conditions [eqn (5)].

## Conclusions

In conclusion, we have developed the first gold-catalyzed oxidative *P*-arylation of *H*-phosphonates promoted by visible light photoredox catalysis. The reaction proceeds under mild reaction conditions (room temperature, no base) and shows excellent substrate scope, including the use of phosphinic acids as coupling partners. More broadly, the use of photoredox catalysis to achieve the oxidation event required for cross-coupling,^5^,^14^ avoids the need for strong oxidants associated with known gold-catalyzed coupling reactions.^15^ This feature putatively allows for increased functional group compatibility, as clearly demonstrated by the gold-catalyzed formation of alkyne-substituted phosphinate ester **3v**, in which the potentially reactive carbon–carbon π-bond^16^ is left intact, and can subsequently be engaged in a copper-catalyzed alkyne-azide click reaction^17^ [eqn (6)]. The development of this strategy for cross-coupling and detailed mechanistic studies is ongoing in our group.
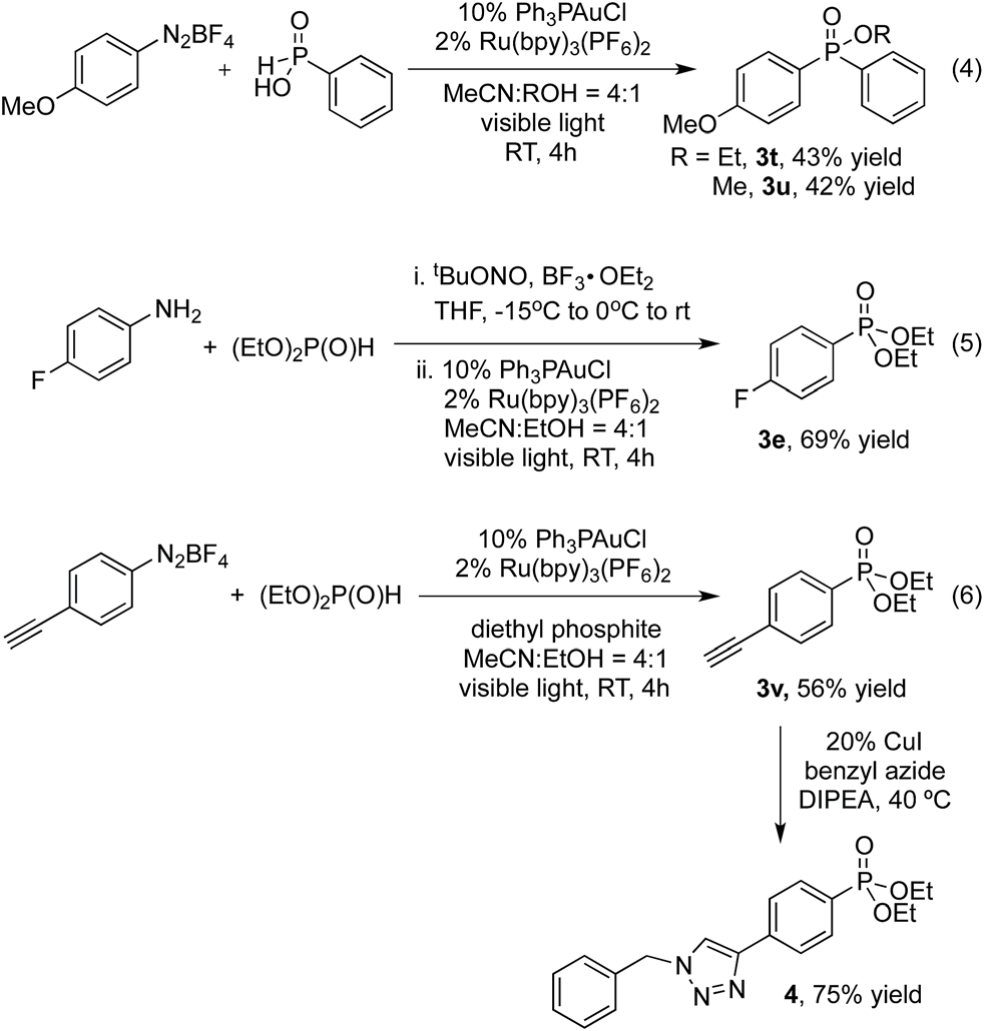


## Supplementary Material

Supplementary information
