# Supplementary material for: A dual catalytic strategy for carbon–phosphorus cross-coupling via gold and photoredox catalysis
Source: Chem Sci. 2014 Nov 25;6(2):1194–8. doi: 10.1039/c4sc03092c (PMC4324597; doi:10.1039/c4sc03092c)
Supplement: Supplementary file 1 [file SC-006-C4SC03092C-s001.pdf]

Electronic Supplementary Material (ESI) for Chemical Science.
This journal is © The Royal Society of Chemistry 2014

A dual catalytic strategy for carbon-phosphorus cross-coupling *via* gold and photoredox catalysis **

Ying He, Hongmiao Wu, and F. Dean Toste*

Table of Contents

1. General Information. 2
2. Preparation of the substrates. 2
3. Optimization of the reaction conditions. 2
4. Effect of various gold catalysts. 3
5. General procedure for P-arylation of H-phosphonate. 4
6. General procedure for the synthesis of 3c (Large Scale). 4
7. General procedure for P-arylation of phenyl phosphinic acid. 5
8. One-pot synthesis of diethyl (4-fluorophenyl)phosphonate (3e). 5
9. Chiral transfer of the diastereoenriched P(O)H compounds. 6
10. General procedure for the synthesis of Diethyl(4-(1-benzyl-1H-1,2,3-triazol-4-yl)phenyl)phosphonate (4) 7
11. Reaction Apparatus 7
12. Characterization data. 8
13. References 14
14. Spectrum Data 15

1. General Information.

Unless otherwise noted, reagents were obtained from commercial sources and used without further purification. All reactions were carried out under N_2 using sealed vial, unless otherwise stated. Dried DMF and THF were obtained by passage through activated alumina columns under argon. Dried acetonitrile was distilled over calcium hydride and stored over 4Å molecular sieves. All other dried solvents used in the reaction were obtained by storage over 3Å or 4Å molecular sieves. TLC analysis of reaction mixtures was performed on Merck silica gel 60 F254 TLC plates and visualized by UV. Preparative TLC was carried out on the same plates. Flash chromatography was carried out with ICN SiliTech 32-63 D 60 Å silica gel. ^{1}H spectra were recorded with Bruker AV-300 or AVQ-400. ^{13}C NMR spectra were recorded with Bruker DRX-500 or AV-600. ^{31}P NMR and ^{19}F NMR spectra were recorded with Bruker AVQ-400. Mass spectral and microanalytical data were obtained via the Micro-Mass/Analytical Facility operated by the College of Chemistry, University of California, Berkeley. The visible light used in reactions was provided by a standard household lamp with a 26 W fluorescent light bulb.

2. Preparation of the substrates.

All the aryl diazonium salts were prepared according to the procedure of Hanson.[1] $[Ru(bpy)_3]_2(PF_6)_2$ (bpy = 2,2'-bipyridine) was prepared according to the procedure of Yoon.[2] Ethyl phenylphosphinate was prepared according to the method of Yu.[3]

3. Optimization of the reaction conditions.

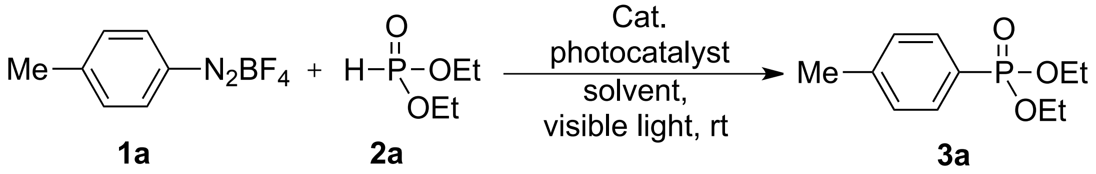

Entry	Cat.	Photocatalyst	Solvent	Time (h)	Yield (%)[b]
1	Ph_3PAuCl	$Ru(bpy)_3(PF_6)_2$	MeCN	4	37
2	Ph_3PAuCl	$Ru(bpy)_3(PF_6)_2$	DMF	4	50
3	Ph_3PAuCl	$Ru(bpy)_3(PF_6)_2$	EtOH	4	65
4	Ph_3PAuCl	$Ru(bpy)_3(PF_6)_2$	DMF : EtOH = 4:1	4	49
5	Ph_3PAuCl	$Ru(bpy)_3(PF_6)_2$	MeCN:EtOH = 4:1	4	82
6	Ph_3PAuCl	$Ru(bpy)_3(PF_6)_2$	MeCN:EtOH = 1:1	4	61
7	Ph_3PAuCl	$Ru(bpy)_3(PF_6)_2$	MeCN:EtOH = 9:1	4	58
8	Ph_3PAuCl	$Ru(bpy)_3Cl_2\cdot 6H_2O$	MeCN:EtOH = 4:1	4	77
9	Ph_3PAuCl	$Ir(ppy)_3$	MeCN:EtOH = 4:1	4	24

10	IPrAuCl	$Ru(bpy)_3(PF_6)_2$	MeCN:EtOH = 4:1	4	0
11[c]	Ph_3PAuCl	$Ru(bpy)_3(PF_6)_2$	MeCN:EtOH = 4:1	4	73
12[d]	Ph_3PAuCl	$Ru(bpy)_3(PF_6)_2$	MeCN:EtOH = 4:1	4	54
13	-	$Ru(bpy)_3(PF_6)_2$	MeCN:EtOH = 4:1	4	0
14	Ph_3PAuCl	-	MeCN:EtOH = 4:1	4	<10
15[e]	Ph_3PAuCl	$Ru(bpy)_3(PF_6)_2$	MeCN:EtOH = 4:1	4	<5
16	$Pd(OAc)_2$	$Ru(bpy)_3(PF_6)_2$	MeCN:EtOH = 4:1	4	43
17	$AgNTf_2$	$Ru(bpy)_3(PF_6)_2$	MeCN:EtOH = 4:1	16	0
18	$AgBF_4$	$Ru(bpy)_3(PF_6)_2$	MeCN:EtOH = 4:1	16	0
19[f]	$AgBF_4$	$Ru(bpy)_3(PF_6)_2$	MeCN:EtOH = 4:1	16	0
20	AgOTf	$Ru(bpy)_3(PF_6)_2$	MeCN:EtOH = 4:1	16	0
21	$Cu(OAc)_2$	$Ru(bpy)_3(PF_6)_2$	MeCN:EtOH = 4:1	16	0
22[g]	$Cu(OAc)_2$	$Ru(bpy)_3(PF_6)_2$	MeCN:EtOH = 4:1	16	0
23	CuI	$Ru(bpy)_3(PF_6)_2$	MeCN:EtOH = 4:1	16	0

[a] Reactions were carried out at room temperature with a 26-W household bulb, 1a (0.3 mmol), 2a (0.1 mmol), Cat. (10 mol%), photocatalyst (2 mol%), degassed solvent (0.5 ml), N_2 atmosphere, rt; [b] Isolated yields; [c] 1 mol% $Ru(bpy)_3(PF_6)_2$ was used; [d] 5 mol% Ph_3PAuCl was used; [e] Without light. [f] 10 mol% PPh_3 was used as the ligand; [g] 10 mol% pyridine was used as the ligand.

4. Effect of various gold catalysts.

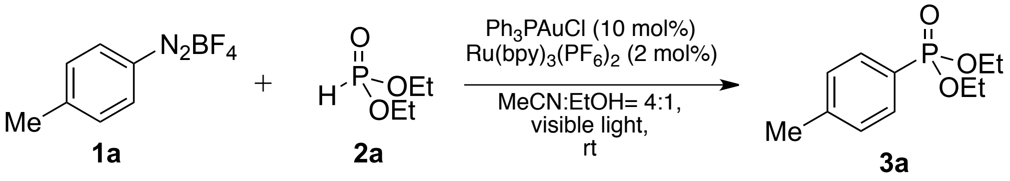

Entry	Cat.	Time (h)	Yield (%)[b]
1	Ph_3PAuCl	4	82
2	IPrAuCl	12	0
3	$dppm(AuCl)_2$	4	78
4	$(4\text{-}CF_3Ph)_3PAuCl$	4	52
5	$(4\text{-}CH_3Ph)_3PAuCl$	4	80
6	Ph_3PAuBr	4	78
7	Me_3PAuCl	4	75

[a] Reactions were carried out at room temperature with a 26-W household bulb, 1a (0.3 mmol), 2a (0.1 mmol), Cat. (10 mol%), photocatalyst (2 mol%), degassed solvent (0.5 ml), N_2 atmosphere, rt; [b] Isolated yields.

5. General procedure for P-arylation of H-phosphonate.

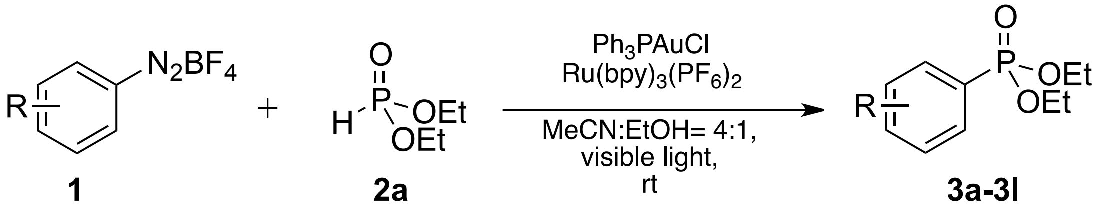

The H-phosphonate ester (0.1 mmol), Ph_3PAuCl (0.01 mmol), $Ru(bpy)_3(PF_6)_2$ (0.002 mmol) and aryldiazonium (0.3 mmol) were added to an oven-dried vial containing a stirring bar. The vial was fitted with a rubber septum. Anhydrous MeCN:EtOH (4:1) (0.5 ml) was added using a syringe in the absence of light, and then the mixture was degassed using freeze-pump-thaw for three cycles (freeze-pump-thaw: cooled to -78°C and degassed via vacuum evacuation for 5 min, backfilled with nitrogen, and warm to room temperature). The reaction stirred with a 26 W household lamp (at 8-10 cm away from the light source) at the room temperature. After the reaction completed, the mixture was quenched with water and aqueous K_2CO_3 solution. The mixture was extracted with DCM, dried with Na_2SO_4, filtered and concentrated in vacuum. The crude product was purified by column chromatography over silica gel using *n*-hexane and ethyl acetate.

6. General procedure for the synthesis of 3c (Large Scale).

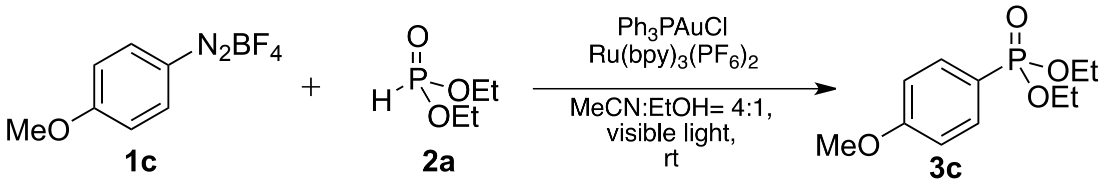

The diethyl phosphite (3.0 mmol), Ph_3PAuCl (8.0 mol%), $Ru(bpy)_3(PF_6)_2$ (2 mol%) and *p*-methoxydiazonium (9.0 mmol) were added to an oven-dried vial containing a stirring bar. The vial was fitted with a rubber septum. Anhydrous MeCN:EtOH (4:1) (15 ml) was added using a syringe in the absence of light, and then the mixture was degassed using freeze-pump-thaw for three cycles (freeze-pump-thaw: cooled to -78°C and degassed via vacuum evacuation for 5 min, backfilled with nitrogen, and warm to room temperature). The reaction stirred with a 26 W household lamp (at 8-10 cm away from the light source) at the room temperature. After the reaction completed, the mixture was quenched with water and aqueous K_2CO_3 solution. The mixture was extracted with DCM, dried with Na_2SO_4, filtered and concentrated in vacuum. The crude product was purified by column chromatography

over silica gel using *n*-hexane and ethyl acetate.

7. General procedure for P-arylation of phenyl phosphinic acid.

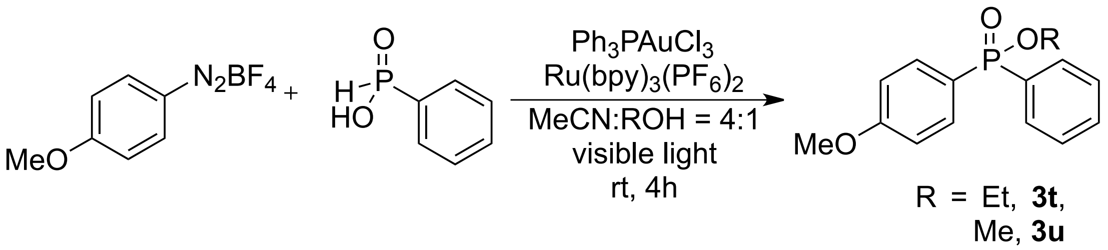

The phenyl phosphinic acid (0.1 mmol), Ph_3PAuCl (0.01 mmol), $Ru(bpy)_3(PF_6)_2$ (0.002 mmol) and aryldiazonium (0.3 mmol) were added to an oven-dried vial containing a stirring bar. The vial was fitted with a rubber septum. Anhydrous MeCN:ROH (R = Me or Et) (4:1) (0.5 ml) was added using a syringe in the absence of light, and then the mixture was degassed using freeze-pump-thaw for three cycles (freeze-pump-thaw: cooled to -78°C and degassed via vacuum evacuation for 5 min, backfilled with nitrogen, and warm to room temperature). The reaction stirred with a 26 W household lamp (at 8-10 cm away from the light source) at the room temperature. After the reaction completed, the mixture was quenched with water and aqueous K_2CO_3 solution. The mixture was extracted with DCM, dried with Na_2SO_4, filtered and concentrated in vacuum. The crude product was purified by column chromatography over silica gel using *n*-hexane and ethyl acetate.

8. One-pot synthesis of diethyl (4-fluorophenyl)phosphonate (3e).

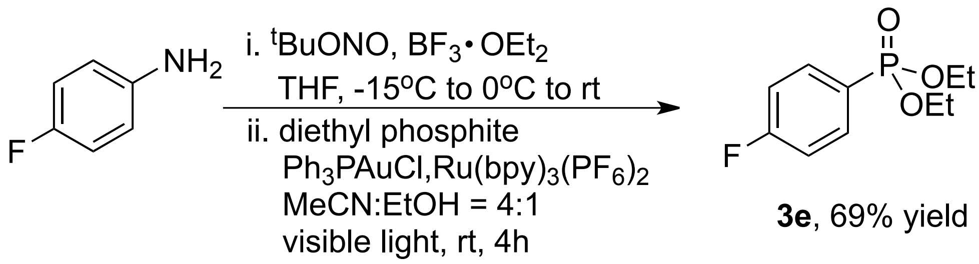

An oven-dried vial was charged with BF_3•OEt_2 (0.33 mmol) in 0.6 ml of anhydrous THF at -15 °C, and then 4-fluoroaniline (0.3 mmol) was added. The vial was sealed and tert-butyl nitrite (0.39 mmol) was added dropwise *via* syringe to the rapidly stirred reaction mixture. After the addition, the mixture was maintained at -15 °C for 10 min and then allowed to warm to 0 °C for 30 min, and room temperature for a certain time which was detected by TLC. Then the reaction mixture was concentrated under reduced pressure. The residue was diluted with anhydrous MeCN:EtOH (4:1) (0.5 ml), and then diethyl phosphite (0.1 mmol), $Ru(bpy)_3(PF_6)_2$

(0.002 mmol) and aryldiazonium (0.3 mmol) were added in the absence of light. The mixture was degassed using freeze-pump-thaw for three cycles (freeze-pump-thaw: cooled to -78 ºC and degassed via vacuum evacuation for 5 min, backfilled with nitrogen, and warm to room temperature). The reaction stirred with a 26 W household lamp (at 8-10 cm away from the light source) at the room temperature. After 4h, the reaction mixture was quenched with water and aqueous K_2CO_3 solution. The mixture was extracted with DCM, dried with Na_2SO_4, filtered and concentrated in vacuum. The crude product was purified by column chromatography over silica gel using *n*-hexane and ethyl acetate.

9. Chirality transfer of the diastereoenriched P(O)H compounds.

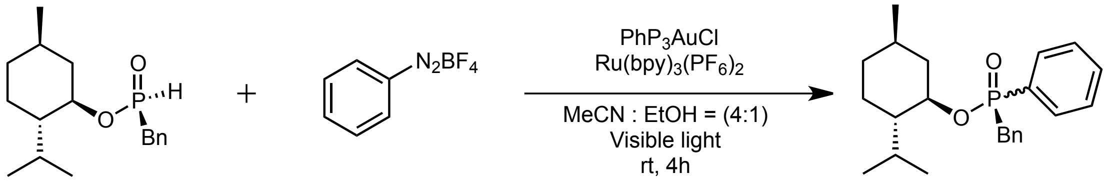

3w
Rp : Sp = 1:1.67

The diastereoenriched P(O)H compound was synthesis according to the literature.[4] The diastereoenriched P(O)H compound (0.1 mmol), Ph_3PAuCl (0.01 mmol), $Ru(bpy)_3(PF_6)_2$ (0.002 mmol) and aryldiazonium (0.3 mmol) were added to an oven-dried vial containing a stirring bar. The vial was fitted with a rubber septum. Anhydrous MeCN:EtOH (4:1) (0.5 ml) was added using a syringe in the absence of light, and then the mixture was degassed using freeze-pump-thaw for three cycles (freeze-pump-thaw: cooled to -78°C and degassed via vacuum evacuation for 5 min, backfilled with nitrogen, and warm to room temperature). The reaction stirred with a 26 W household lamp (at 8-10 cm away from the light source) at the room temperature. After the reaction completed, the mixture was quenched with water and aqueous K_2CO_3 solution. The mixture was extracted with DCM, dried with Na_2SO_4, filtered and concentrated in vacuum. The *dr* ratio of the **3w** was determined by ^{31}P NMR.

10. General procedure for the synthesis of Diethyl(4-(1-benzyl-1H-1, 2,3-triazol-4-yl)phenyl)phosphonate (4)

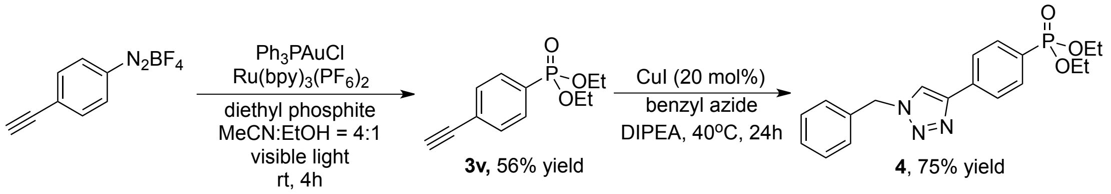

The diethyl (4-(prop-1-yn-1-yl)phenyl)phosphonate (**3v**) was synthesized according to the procedure in section **4** described above. The **3v** (0.1 mmol), CuI (0.02 mmol), N, N-Diisopropylethylamine (DIPEA) (0.1 mmol) and benzyl azide (0.1 mmol) was charged with anhydrous MeOH in an oven-dried vial. The mixture was stirred at 40 °C for 24 h. The reaction mixture was then concentrated under reduced pressure. The crude product was purified by column chromatography over silica gel using *n*-hexane and ethyl acetate.

11. Reaction Apparatus

Photochemical reaction was carried out under visible light irradiation by a PHILIPS 26 W fluorescent bulb (approximately 8-10 cm from the reaction) at room temperature.

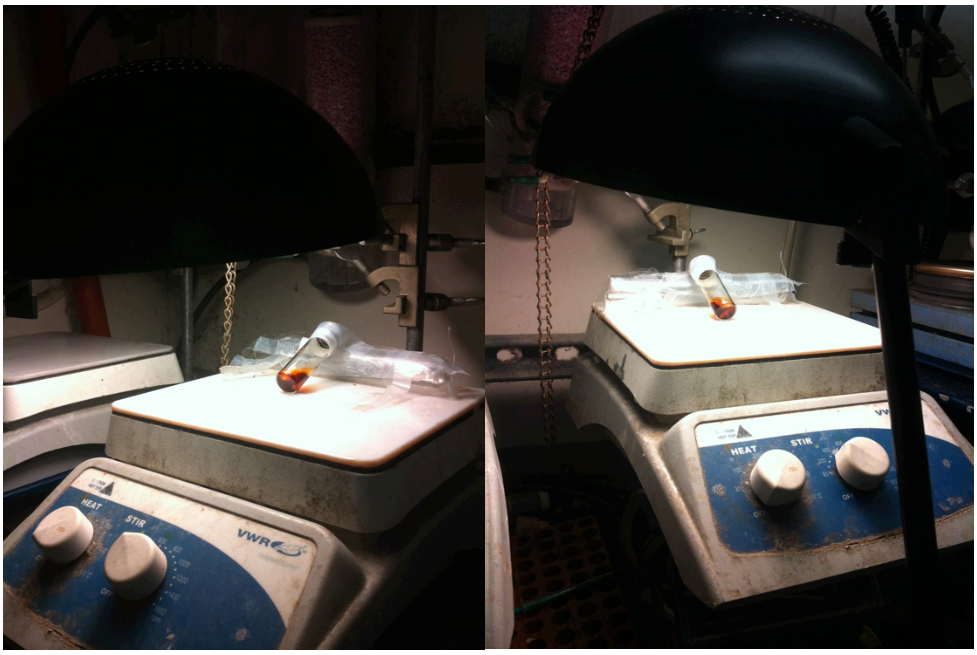

12. Characterization data.

Some of the products are known compounds and the spectral data matched that previously reported[4-7].

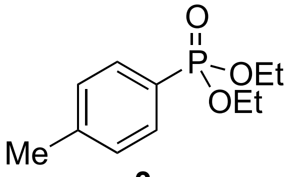

3a

Diethyl *p*-tolylphosphonate (**3a**): oil, ^{1}H NMR (400 MHz) ($CDCl_3$): δ 7.70 (dd, J_1 = 13.2 Hz, J_2 = 8.0 Hz, 2H), 7.27-7.29 (m, 2H), 4.05-4.15 (m, 4H), 2.41 (s, 3H), 1.32 (t, J = 7.2 Hz, 6H).

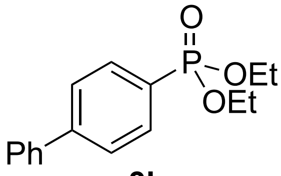

3b

Diethyl [1,1'-biphenyl]-4-ylphosphonate (**3b**): oil, ^{1}H NMR (300 MHz) ($CDCl_3$): δ 7.88 (dd, J_1 = 13.2 Hz, J_2 = 8.4 Hz, 2H), 7.69 (dd, J_1 = 8.1 Hz, J_2 = 3.9 Hz, 2H), 7.59-7.62 (m, 2H), 7.39-7.49 (m, 3H), 4.06-4.21 (m, 4H), 1.34 (t, J = 7.2 Hz, 6H).

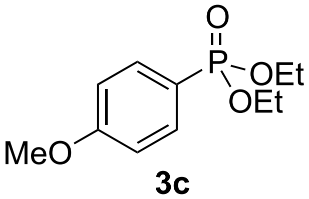

3c

Diethyl (4-methoxyphenyl)phosphonate (**3c**): oil, ^{1}H NMR (300 MHz) ($CDCl_3$): δ 7.73 (dd, J_1 = 12.9 Hz, J_2 = 9.0 Hz, 2H), 6.95 (dd, J_1 = 8.7 Hz, J_2 = 3.3 Hz, 2H), 4.02-4.11 (m, 4H), 3.83 (s, 3H), 1.29 (t, J = 7.2 Hz, 6H).

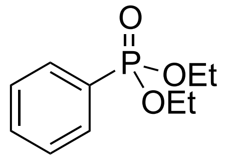

3d

Diethyl phenylphosphonate (**3d**): oil, ^{1}H NMR (300 MHz) ($CDCl_3$): δ 7.74-7.79 (m, 2H), 7.40-7.52 (m, 3H), 4.00-4.16 (m, 4H), 1.29 (t, J = 7.2 Hz, 6H).

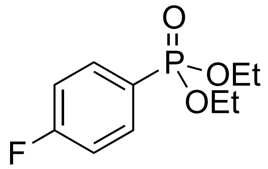

3e

Diethyl (4-fluorophenyl)phosphonate (**3e**): oil, ^{1}H NMR (300 MHz) ($CDCl_3$): δ 7.76-7.85 (m, 2H), 7.10-7.17 (m, 2H), 4.04-4.15 (m, 4H), 1.30 (t, J = 7.2 Hz, 6H).

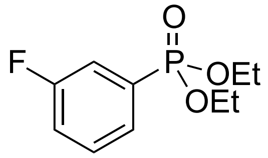

3f

Diethyl (3-fluorophenyl)phosphonate (**3f**): oil, ^{1}H NMR (300 MHz) ($CDCl_3$): δ 7.41-7.62 (m, 3H), 7.20-7.25 (m, 1H), 4.04-4.19 (m, 4H), 1.32 (t, J = 7.2 Hz, 6H).

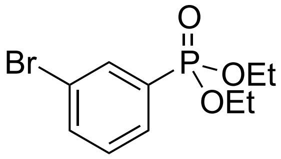

3g

Diethyl (3-bromophenyl)phosphonate (**3g**): oil, ^{1}H NMR (300 MHz) ($CDCl_3$): δ 7.94 (d, J = 14.0 Hz, 1H), 7.65-7.90 (m, 2H), 7.31-7.37 (m, 1H), 4.03-4.19 (m, 4H), 1.32 (t, J = 7.2 Hz, 6H).

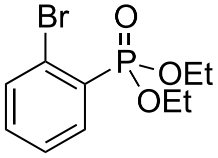

3h

Diethyl (2-bromophenyl)phosphonate: oil (**3h**), ^{1}H NMR (400 MHz) ($CDCl_3$): δ 8.00-8.05 (m, 1H), 7.66-7.69 (m, 1H), 7.38-7.41 (m, 2H), 4.11-4.24 (m, 4H), 1.37 (t, J = 7.2 Hz, 6H).

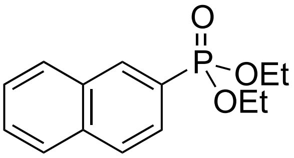

3i

Diethyl naphthalen-1-ylphosphonate: oil (**3i**), ^{1}H NMR (400 MHz) ($CDCl_3$): δ 8.52 (d, J = 8.4 Hz, 1H), 8.26 (dd, J_1 = 16.0 Hz, J_2 = 6.8 Hz, 1H), 8.04-8.06 (d, 1H), 7.90-7.92 (d, 1H), 7.52-7.64 (m, 3H), 4.04-4.13 (m, 2H), 4.17-4.27 (m, 2H), 1.31 (t, J = 7.2 Hz, 6H).

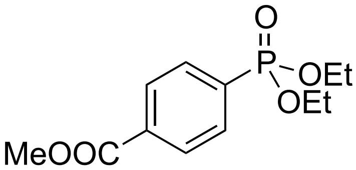

3j

Ethyl 4-(diethoxyphosphoryl)benzoate (**3j**): oil, ^{1}H NMR (300 MHz) ($CDCl_3$): δ 8.09-8.13 (m, 2H), 7.41-7.91 (m, 2H), 4.04-4.20 (m, 4H), 3.93 (s, 3H), 1.32 (t, J = 7.2 Hz, 6H).

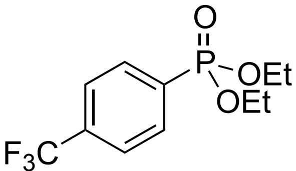

3k

Diethyl (4-(trifluoromethyl)phenyl)phosphonate (**3k**): oil, ^{1}H NMR (300 MHz) ($CDCl_3$): δ 7.93 (dd, J_1 = 13.2 Hz, J_2 = 8.1 Hz, 2H), 7.70-7.74 (m, 2H), 4.05-4.21 (m, 4H), 1.32 (t, J = 7.2 Hz, 6H).

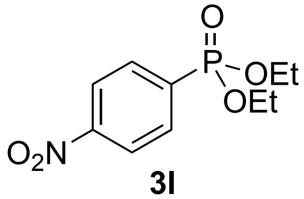

3l

Diethyl (4-nitrophenyl)phosphonate (**3l**): ^{1}H NMR (300 MHz) ($CDCl_3$): δ 8.30 (dd, J_1 = 6.9 Hz, J_2 = 3.3 Hz, 2H), 7.99 (dd, J_1 = 12.6 Hz, J_2 = 8.7 Hz, 2H), 4.10-4.21 (m, 4H), 1.34 (t, J = 7.2 Hz, 6H).

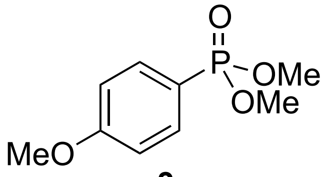

3m

Dimethyl (4-methoxyphenyl)phosphonate (**3m**): oil, ^{1}H NMR (300 MHz) ($CDCl_3$): δ 7.71 (dd, J_1 = 12.9 Hz, J_2 = 9.0 Hz, 2H), 6.93-6.97 (m, 2H), 3.83 (s, 3H), 3.71 (d, J = 11.1 Hz, 6H).

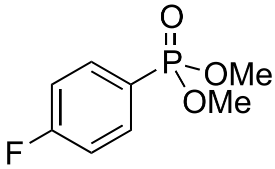

3n

Dimethyl (4-fluorophenyl)phosphonate (**3n**): oil, ^{1}H NMR (400 MHz) ($CDCl_3$): δ 7.79-7.85 (m, 2H), 7.15-7.20 (m, 2H), 3.77 (d, J = 11.2 Hz, 6H).

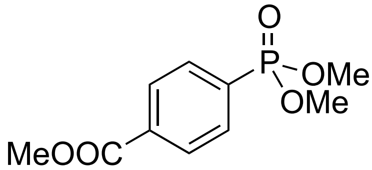

3o

Methyl 4-(dimethoxyphosphoryl)benzoate (**3o**): oil, ^{1}H NMR (400 MHz) ($CDCl_3$): δ 8.11-8.14 (m, 2H), 7.85-7.90 (m, 2H), 3.94 (s, 3H), 3.78 (d, J = 10.8 Hz, 6H).

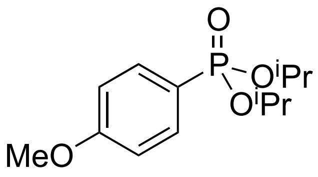

3p

Diisopropyl (4-methoxyphenyl)phosphonate (**3p**): oil, ^{1}H NMR (300 MHz) ($CDCl_3$): δ 7.73 (dd, J_1 = 12.9 Hz, J_2 = 9.0 Hz, 2H), 6.93 (dd, J_1 = 8.7 Hz, J_2 = 3.3 Hz, 2H), 4.59-4.66 (m, 2H), 3.83 (s, 3H), 1.34 (d, J = 6.3 Hz, 6H), 1.19 (d, J = 6.3 Hz, 6H).

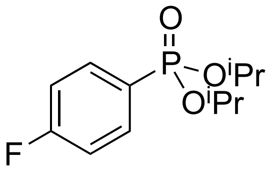

3q

Diisopropyl (4-fluorophenyl)phosphonate (**3q**): oil, ^{1}H NMR (400 MHz) ($CDCl_3$): δ 7.79-7.86 (m, 2H), 7.12-7.16 (m, 2H), 4.65-4.73 (m, 2H), 1.38 (d, J = 6.4 Hz, 6H), 1.23 (d, J = 6.0 Hz, 6H); ^{13}C NMR (150 MHz) ($CDCl_3$): δ 165.1 (dd, J_1 = 4.5 Hz, J_2 = 252 Hz), 134.2 (dd, J_1 = 10.5 Hz, J_2 = 9.0 Hz), 126.1 (dd, J_1 = 193.5 Hz, J_2 = 4.5 Hz), 115.6 (dd, J_1 = 16.5 Hz, J_2 = 21.0 Hz), 70.8 (d, J = 4.5 Hz), 23.9 (dd, J_1 = 33.0 Hz, J_2 = 4.5 Hz); ^{31}P NMR (162 MHz) δ 15.6; ^{19}F NMR (376 MHz) δ -105.8. HRMS (ESI) calcd. for $C_{12}H_{18}FO_3P$ $(M + H)^+$: 261.1056, Found: 261.1055.

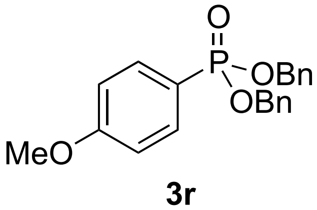

3r

Dibenzyl (4-methoxyphenyl)phosphonate (**3r**): oil, ^{1}H NMR (400 MHz) ($CDCl_3$): δ 7.75 (dd, J_1 = 12.9 Hz, J_2 = 8.7 Hz, 2H), 7.25-7.33 (m, 10H), 6.93 (dd, J_1 = 9.0 Hz, J_2 = 3.6 Hz, 2H), 4.97-5.11 (m, 4H), 3.84 (s, 3H); ^{13}C NMR (125 MHz) ($CDCl_3$): δ 163.0 (d, J = 3.75), 136.3 (d, J = 7.5 Hz), 133.9 (d, J = 11.25 Hz), 128.5, 128.2, 127.9, 119.0 (d, J = 196.3 Hz), 114.1 (d, J_1 = 16.25 Hz), 67.4 (d, J = 50 Hz), 55.4; ^{31}P NMR (162 MHz) δ 20.6. HRMS (ESI) calcd. for $C_{21}H_{21}O_4P$ $(M + H)^+$: 369.1256, Found: 369.1259.

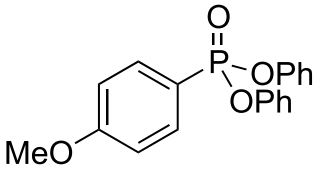

3s

Diphenyl (4-methoxyphenyl)phosphonate: oil (**3s**), ^{1}H NMR (300 MHz) ($CDCl_3$): δ 7.88 (dd, J_1 = 13.2 Hz, J_2 = 8.7 Hz, 2H), 7.25-7.31 (m, 5H), 7.13-7.19 (m, 5H), 6.98 (dd, J_1 = 8.7 Hz, J_2 = 3.6 Hz, 2H), 3.85 (s, 3H).

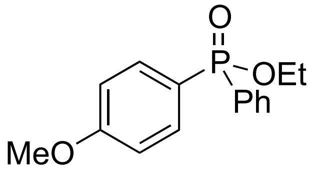

3t

Ethyl (4-methoxyphenyl)(phenyl)phosphinate (**3t**): oil, ^{1}H NMR (400 MHz) ($CDCl_3$): δ 7.73-7.82 (m, 4H), 7.43-7.50 (m, 3H), 6.96 (dd, J_1 = 8.8 Hz, J_2 = 2.4 Hz, 2H), 4.05-4.13 (m, 2H), 3.83 (s, 3H), 1.36 (t, J = 6.8 Hz, 3H); ^{13}C NMR (125 MHz) ($CDCl_3$): δ 162.7, 133.6 (d, J = 11.3 Hz), 132.1 (d, J = 10.0 Hz), 131.9 (d, J = 2.5 Hz), 131.5 (d, J = 10.0 Hz), 128.5 (d, J = 12.5 Hz), 122.7 (d, J = 143.8 Hz), 114.1 (d, J = 13.8 Hz), 61.0 (d, J = 6.3 Hz), 55.35, 16.5 (d, J = 7.5 Hz); ^{31}P NMR (162 MHz) δ 31.7. HRMS (ESI) calcd. for $C_{15}H_{17}O_3P$ $(M + H)^+$: 277.0994, Found: 277.0994.

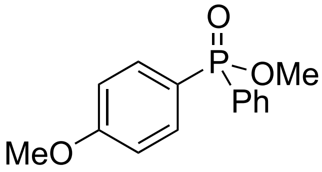

3u

Methyl (4-methoxyphenyl)(phenyl)phosphinate (**3u**): oil, ^{1}H NMR (400 MHz) ($CDCl_3$): δ 7.66-7.82 (m, 4H), 7.46-7.54 (m, 3H), 6.98 (dd, J_1 = 8.8 Hz, J_2 = 2.8 Hz, 2H), 3.85 (s, 3H), 3.75 (d, J = 11.2 Hz, 3H); ^{13}C NMR (125 MHz) ($CDCl_3$): δ 162.8, 133.7 (d, J = 11.3 Hz), 132.0 (d, J = 3.8 Hz), 131.5 (d, J = 10.0 Hz), 131.0, 128.5 (d, J = 13.8 Hz), 122.5 (d, J = 142.5 Hz), 114.1 (d, J = 15.0 Hz), 55.4, 51.5 (d, J = 6.25 Hz); ^{31}P NMR (162 MHz) δ 33.6. ^{31}P NMR (162 MHz) δ 31.7. HRMS (ESI) calcd. for $C_{14}H_{15}O_3P$ $(M + H)^+$: 263.0837, Found: 263.0837.

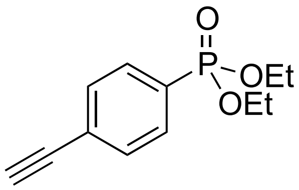

3v

Diethyl (4-(prop-1-yn-1-yl)phenyl)phosphonate (**3v**): oil, ^{1}H NMR (300 MHz) ($CDCl_3$): δ 7.72-7.77 (m, 2H), 7.54-7.58 (m, 2H), 4.06-4.16 (m, 4H), 3.12 (s, 1H), 1.31 (t, J = 7.2 Hz, 6H).

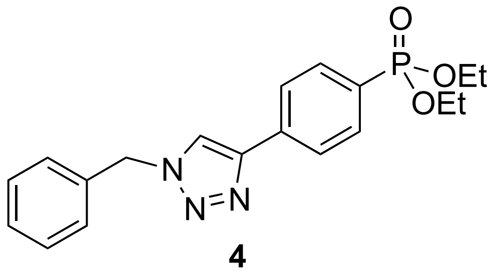

4

Diethyl (4-(1-benzyl-1*H*-1,2,3-triazol-4-yl)phenyl)phosphonate (**4**): oil, ^{1}H NMR (400 MHz) ($CDCl_3$): δ 7.82-7.92 (m, 4H), 7.75 (s, 1H), 7.27-7.41 (m, 5H), 5.60 (s, 2H), 4.05-4.18 (m, 4H), 1.32 (t, J = 6.8, 6H); ^{13}C NMR (125 MHz) ($CDCl_3$): δ 147.1, 134.4, 132.4 (d, J = 10.0 Hz), 129.3, 129.0, 128.6, 128.2, 127.1, 125.6 (d, J = 15.0 Hz), 120.4, 62.2 (d, J = 5.0 Hz), 54.4, 16.3 (d, J = 7.5 Hz); ^{31}P NMR (162 MHz) δ 18.5. ^{31}P NMR (162 MHz) δ 31.7. HRMS (ESI) calcd. for $C_{19}H_{22}N_3O_3P$ $(M + H)^+$: 372.1477, Found: 372.1478.

13. References

[1] P. Hanson, J. R. Jones, A. B. Taylor, P. H. Walton, A. W. Timms, *J. Chem. Soc. Perkin Trans. 2.* **2002**, 1135-1150.
[2] M. A. Ishay, Z. Lu, T. P. Yoon, *J. Am. Chem. Soc.* **2010**, *132*, 8572-8574.
[3] K. Afarinkia, H. W. Yu, *Tetrahedron Lett.* **2003**, *44*, 781-783.
[4] Q. Xu, C. Q. Zhao, L. B. Han, *J. Am. Chem. Soc*. **2008**, *130*, 12648-12655.
[5] R. Berrino, S. Cacchi, G. Fabrizi, A. Goggiamani, P. Stabile, *Org. Biomol. Chem.* **2010**, *8*, 4518-4520.
[6] R. Q. Zhuang, J. Xu, Z. S. Cai, G. Tang, M. J. Fang, Y. F. Zhao, *Org. Lett*. **2011**, *13*, 2110-2113.
[7] H. Onouchi, K. Maeda, E. Yashima, *J. Am. Chem. Soc*. **2001**, *123*, 7441-7442.

14. Spectrum Data

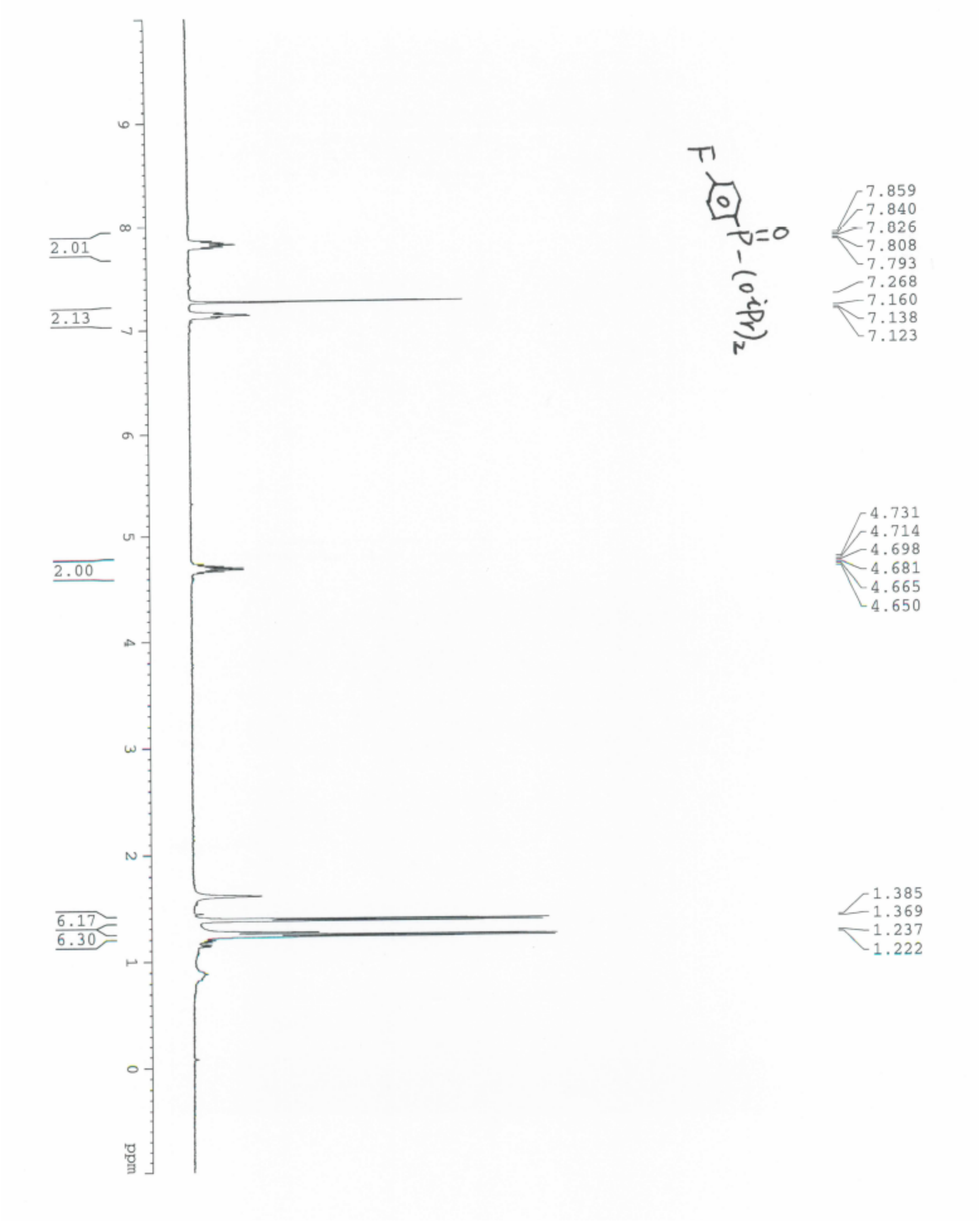

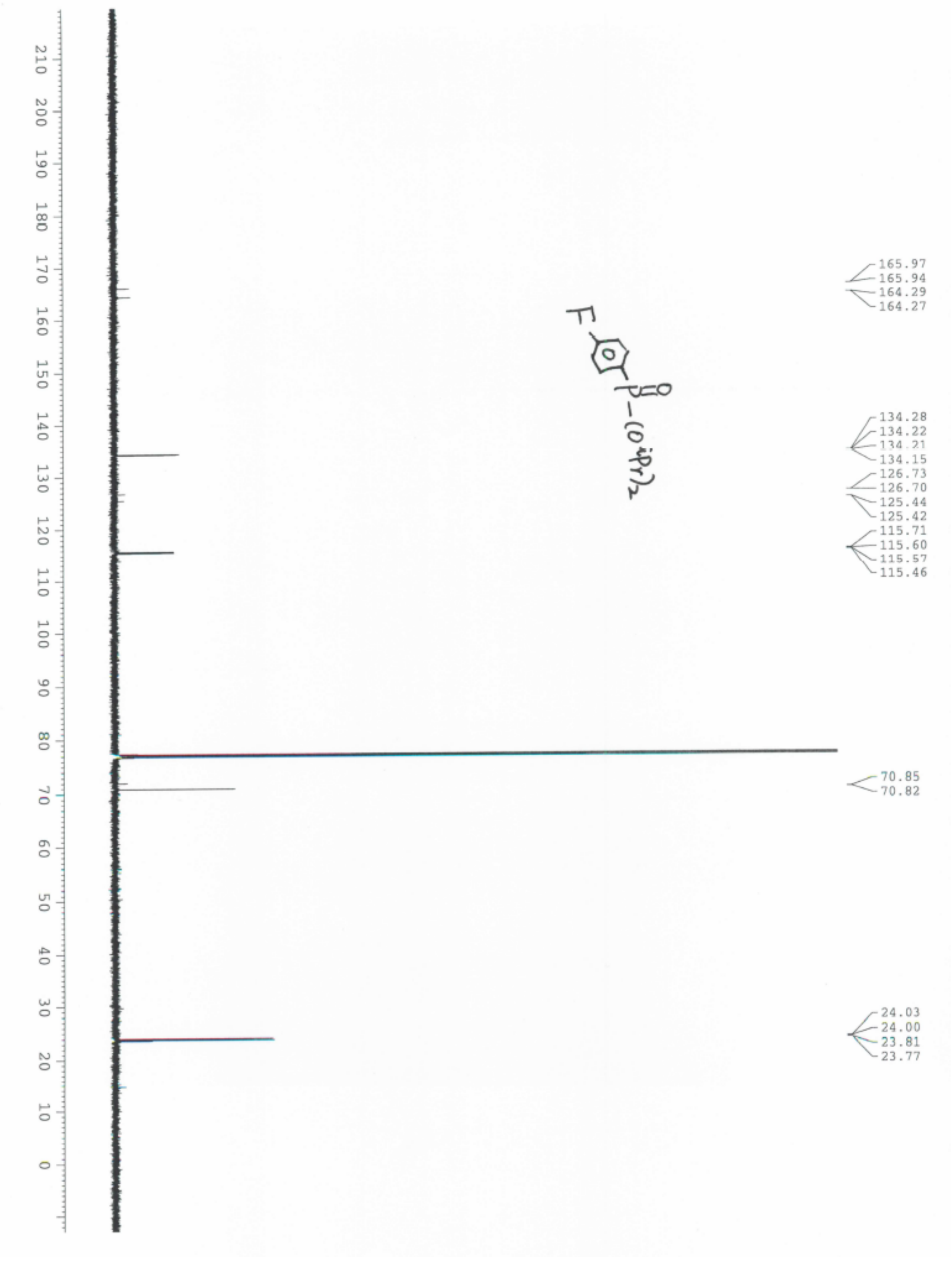
165.97
165.94
164.29
164.27
134.28
134.22
134.21
134.15
126.73
126.70
125.44
125.42
115.71
115.60
115.57
115.46
70.85
70.82
24.03
24.00
23.81
23.77
210 200 190 180 170 160 150 140 130 120 110 100 90 80 70 60 50 40 30 20 10 0

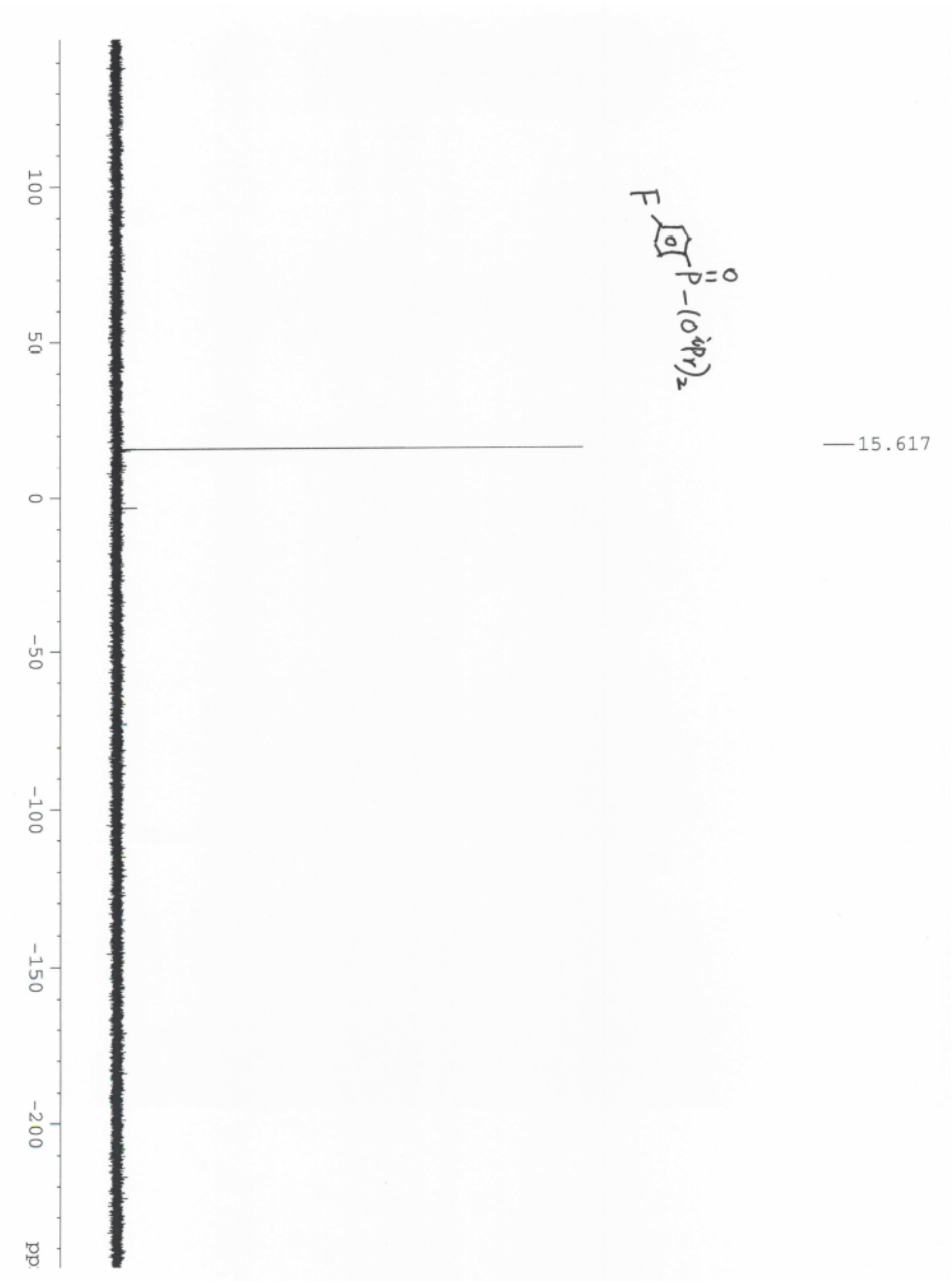
15.617
100
50
0
-50
-100
-150
-200
pp

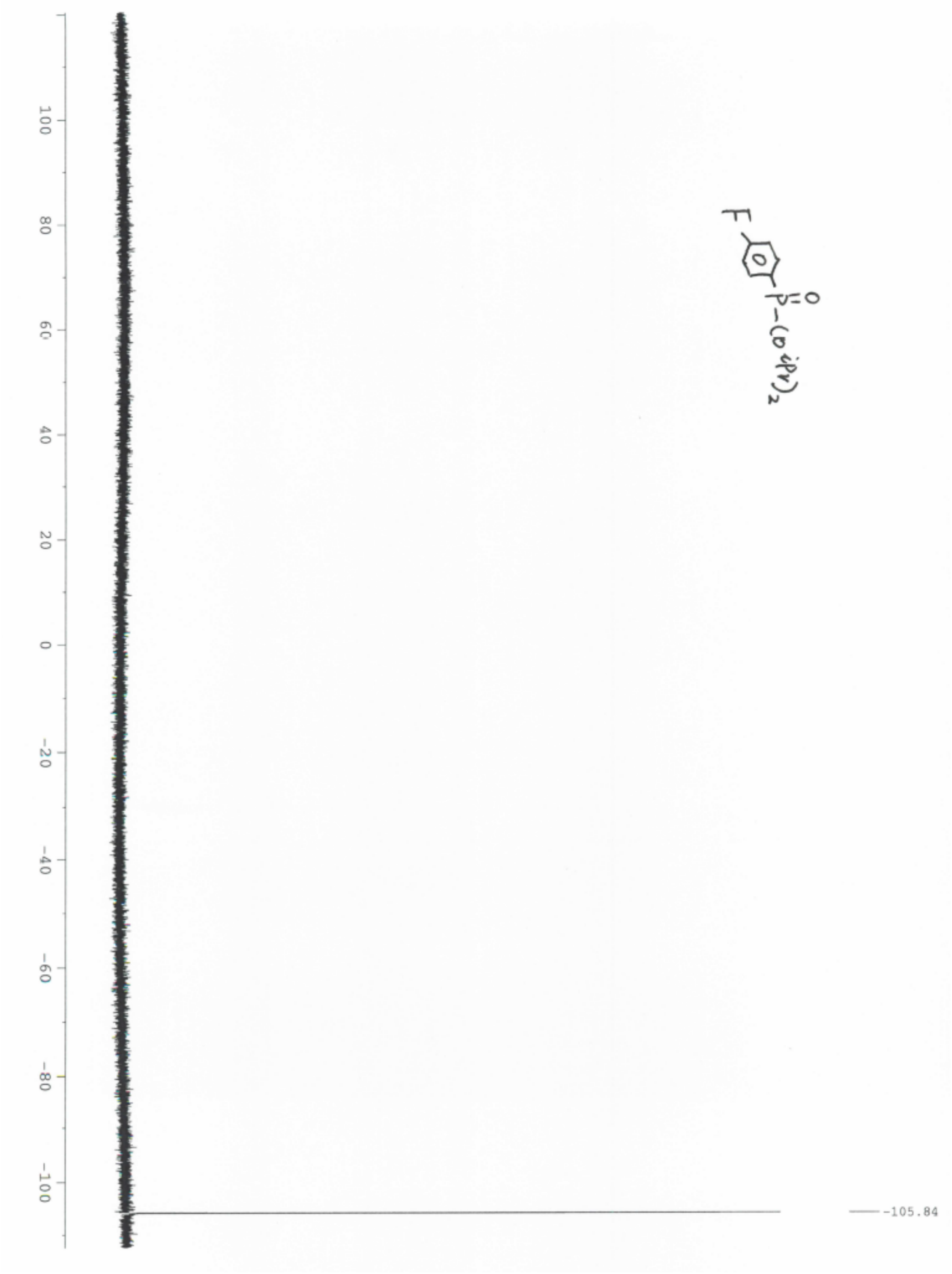

100
80
60
40
20
0
-20
-40
-60
-80
-100
-105.84

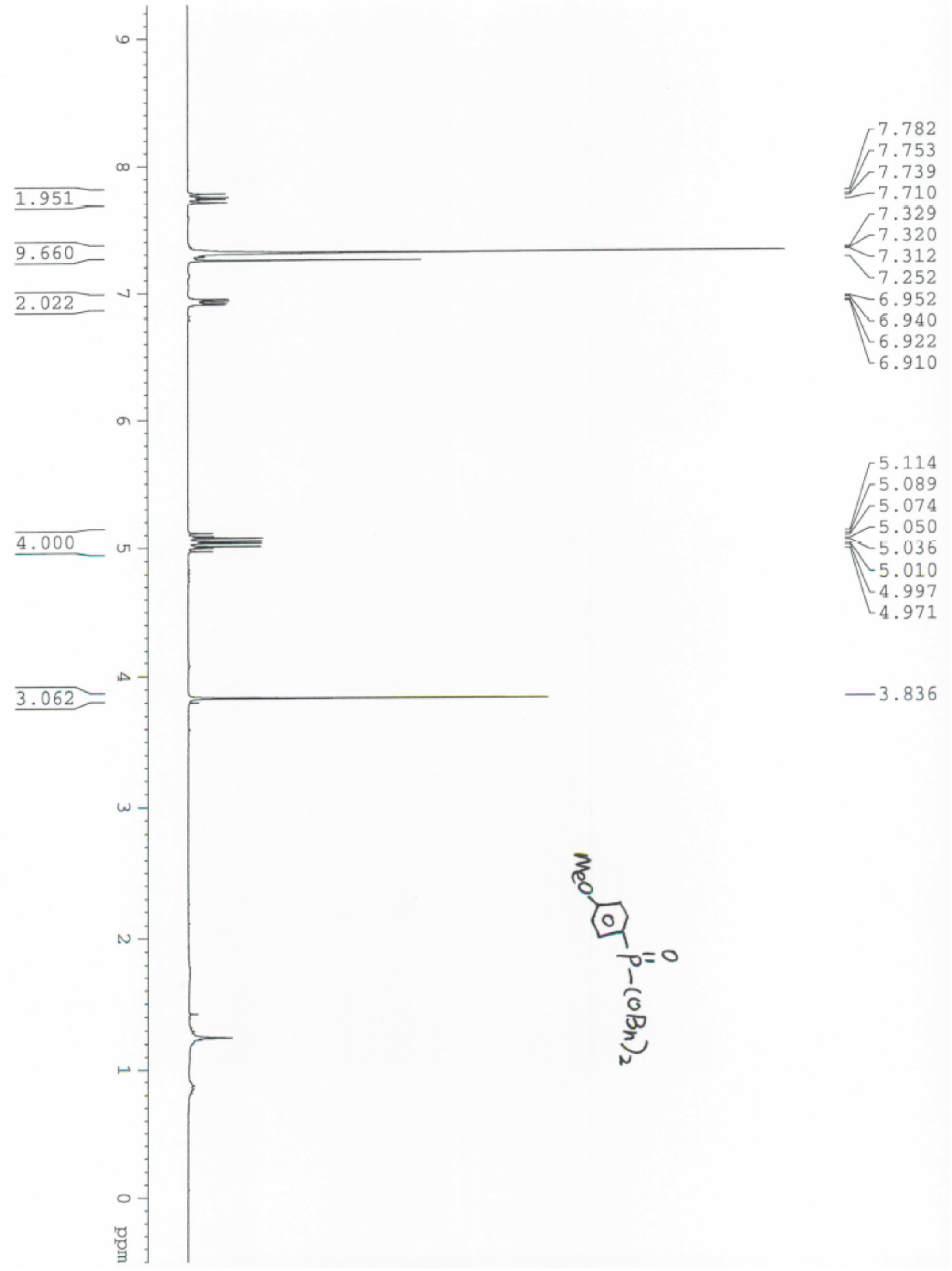
7.782
7.753
7.739
7.710
7.329
7.320
7.312
7.252
6.952
6.940
6.922
6.910
5.114
5.089
5.074
5.050
5.036
5.010
4.997
4.971
3.836
1.951
9.660
2.022
4.000
3.062
ppm
MeO
P-(OBn)2

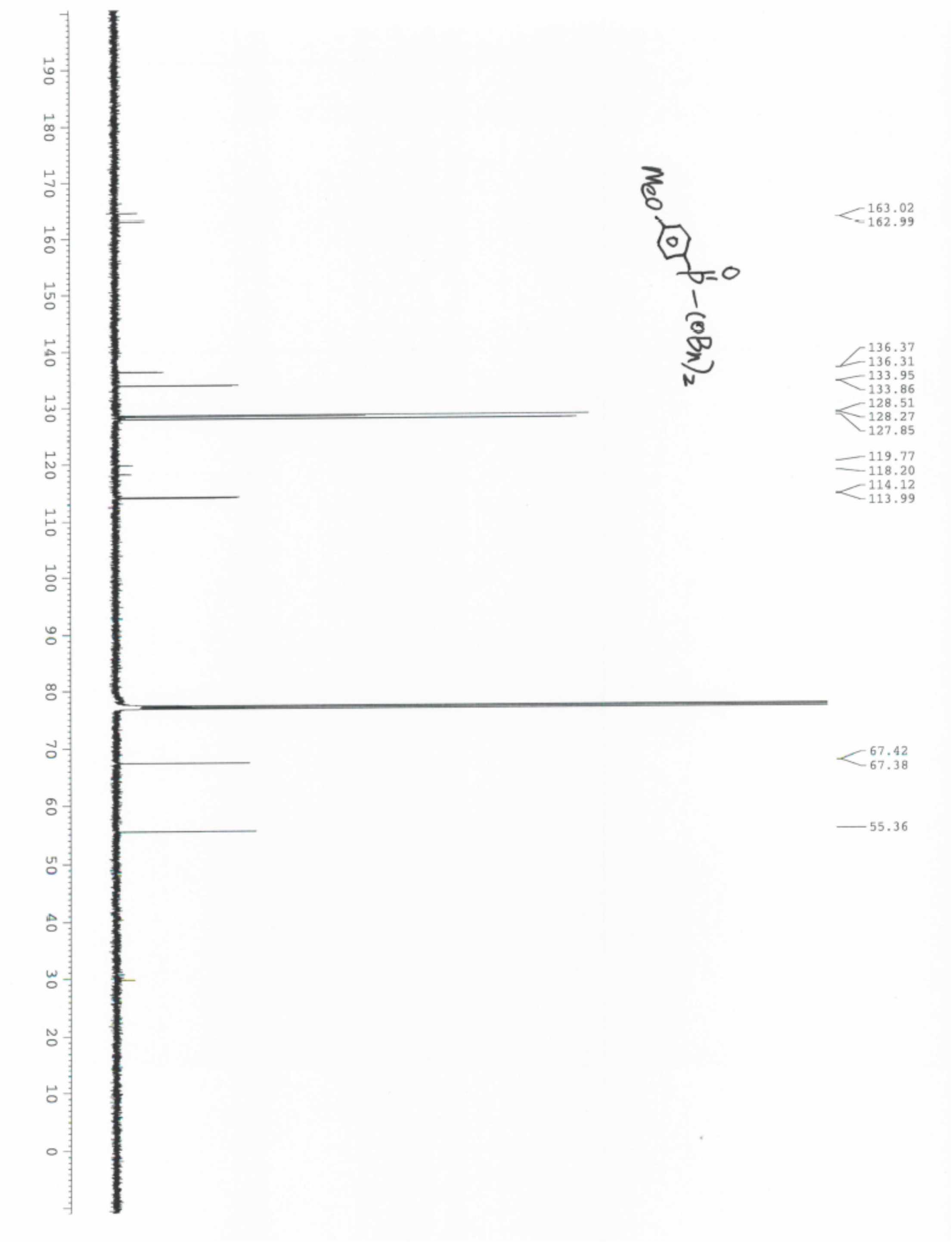
163.02
162.99
136.37
136.31
133.95
133.86
128.51
128.27
127.85
119.77
118.20
114.12
113.99
67.42
67.38
55.36

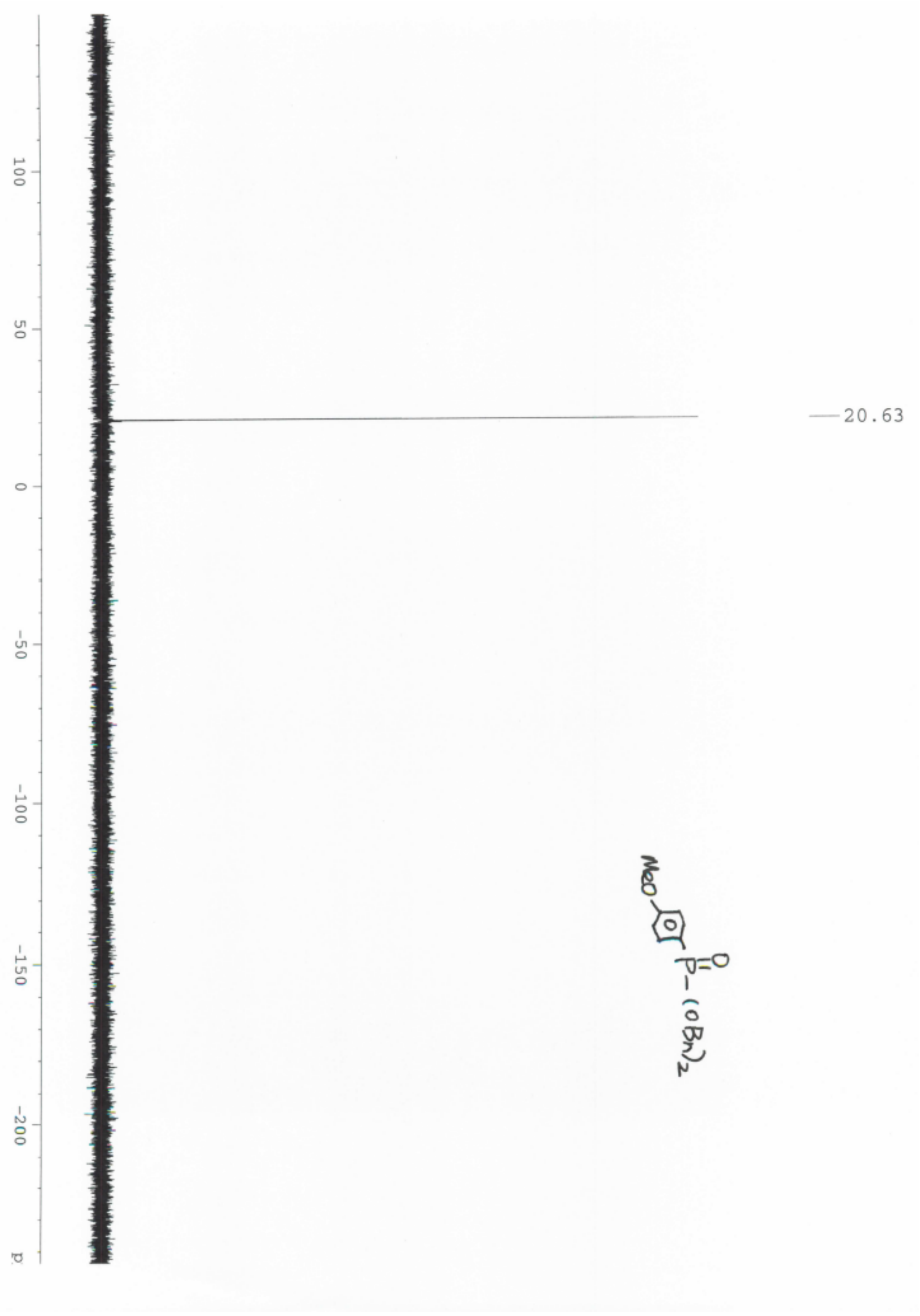
100
50
0
-50
-100
-150
-200
20.63

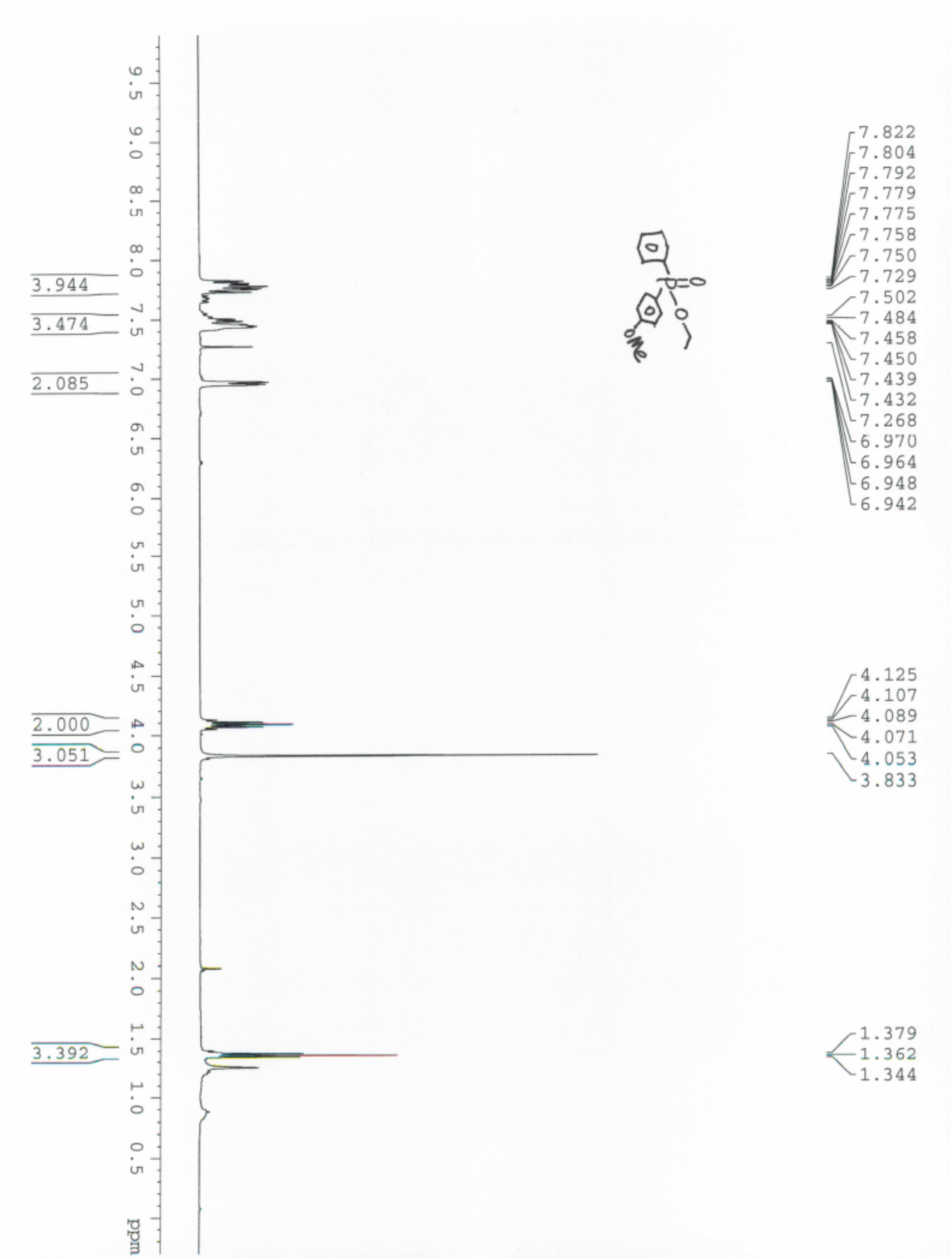
7.822
7.804
7.792
7.779
7.775
7.758
7.750
7.729
7.502
7.484
7.458
7.450
7.439
7.432
7.268
6.970
6.964
6.948
6.942
4.125
4.107
4.089
4.071
4.053
3.833
1.379
1.362
1.344
3.944
3.474
2.085
2.000
3.051
3.392
ppm

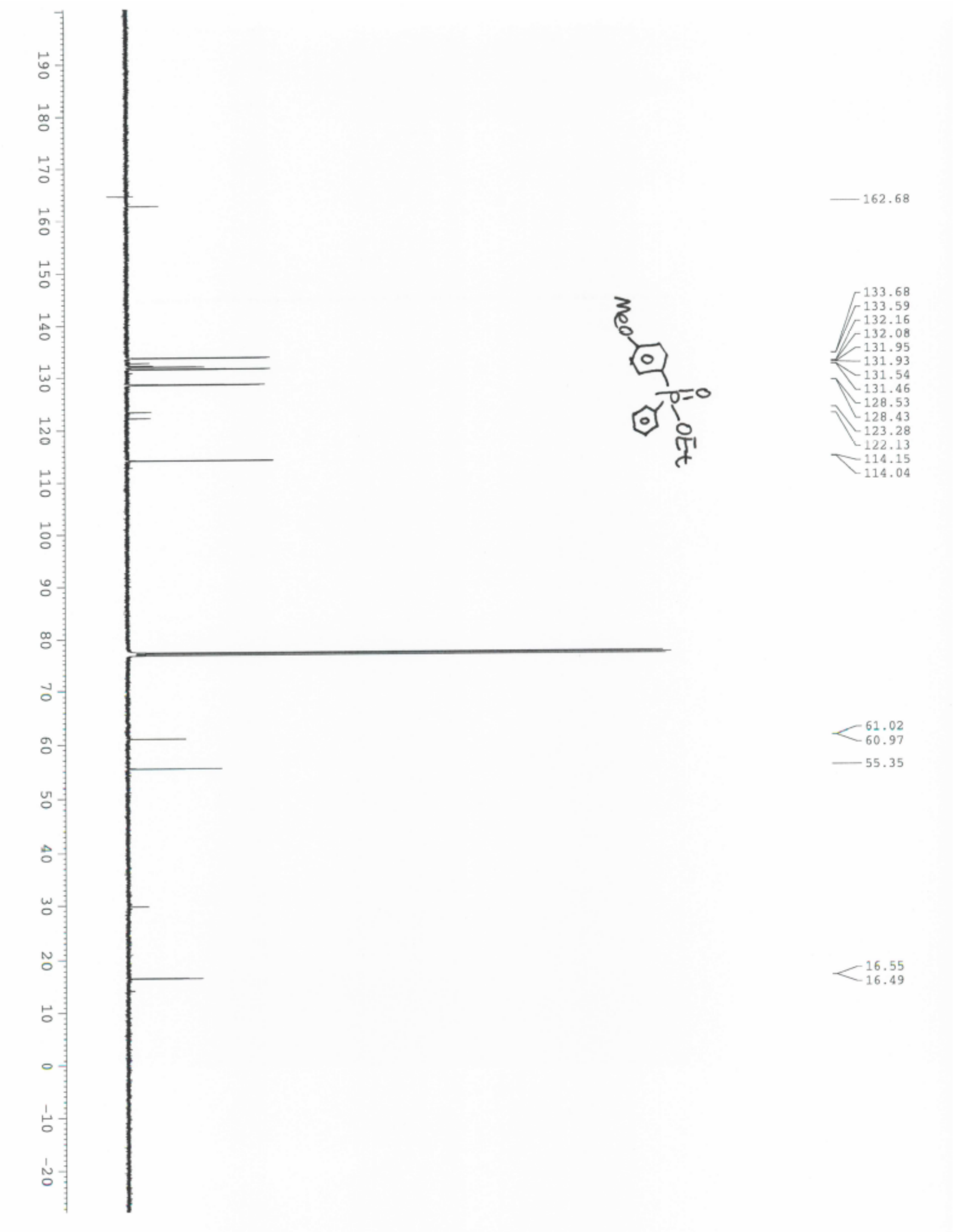
162.68
133.68
133.59
132.16
132.08
131.95
131.93
131.54
131.46
128.53
128.43
123.28
122.13
114.15
114.04
61.02
60.97
55.35
16.55
16.49
MeO
OEt

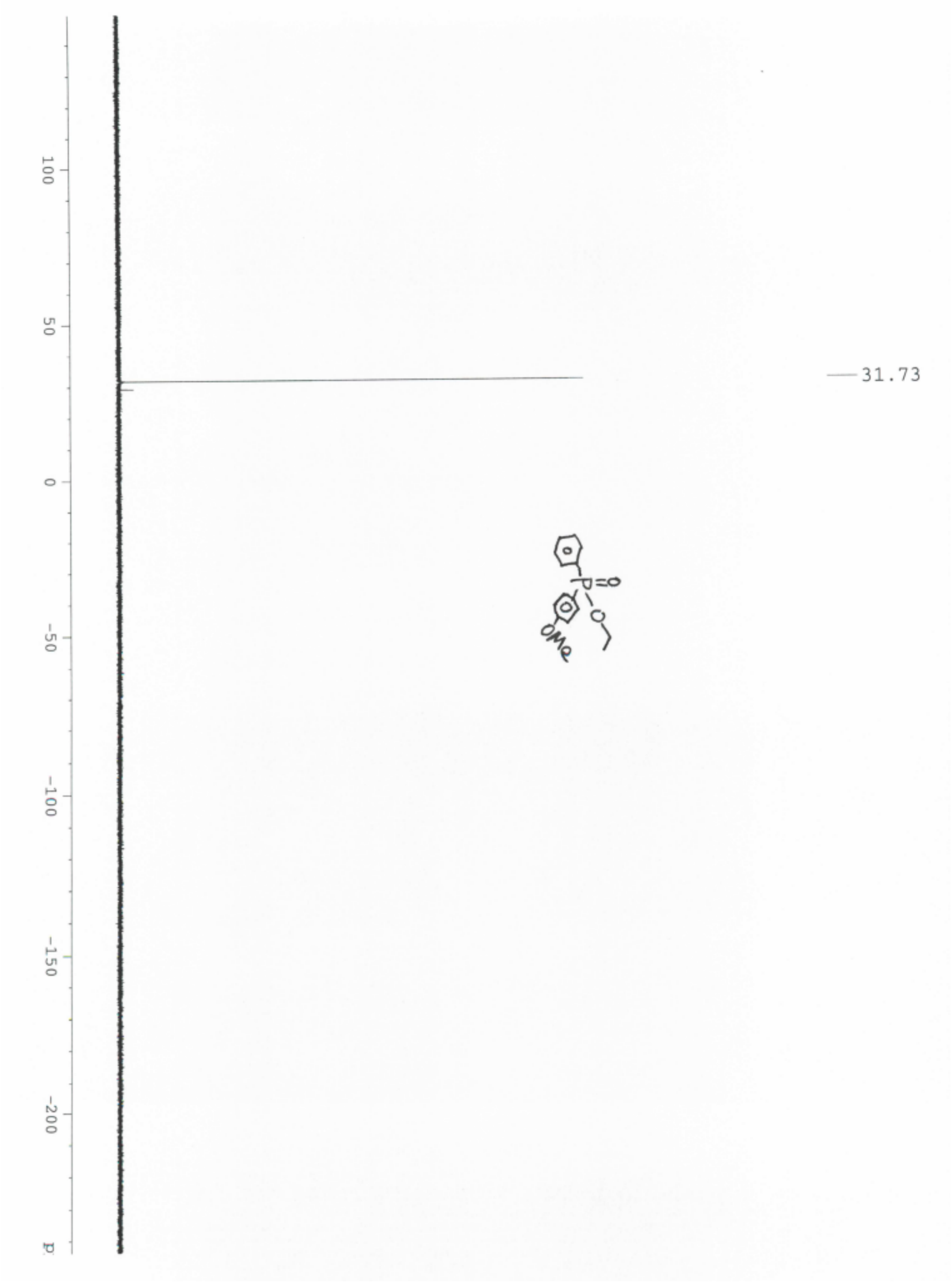
31.73
100
50
0
-50
-100
-150
-200
OMe

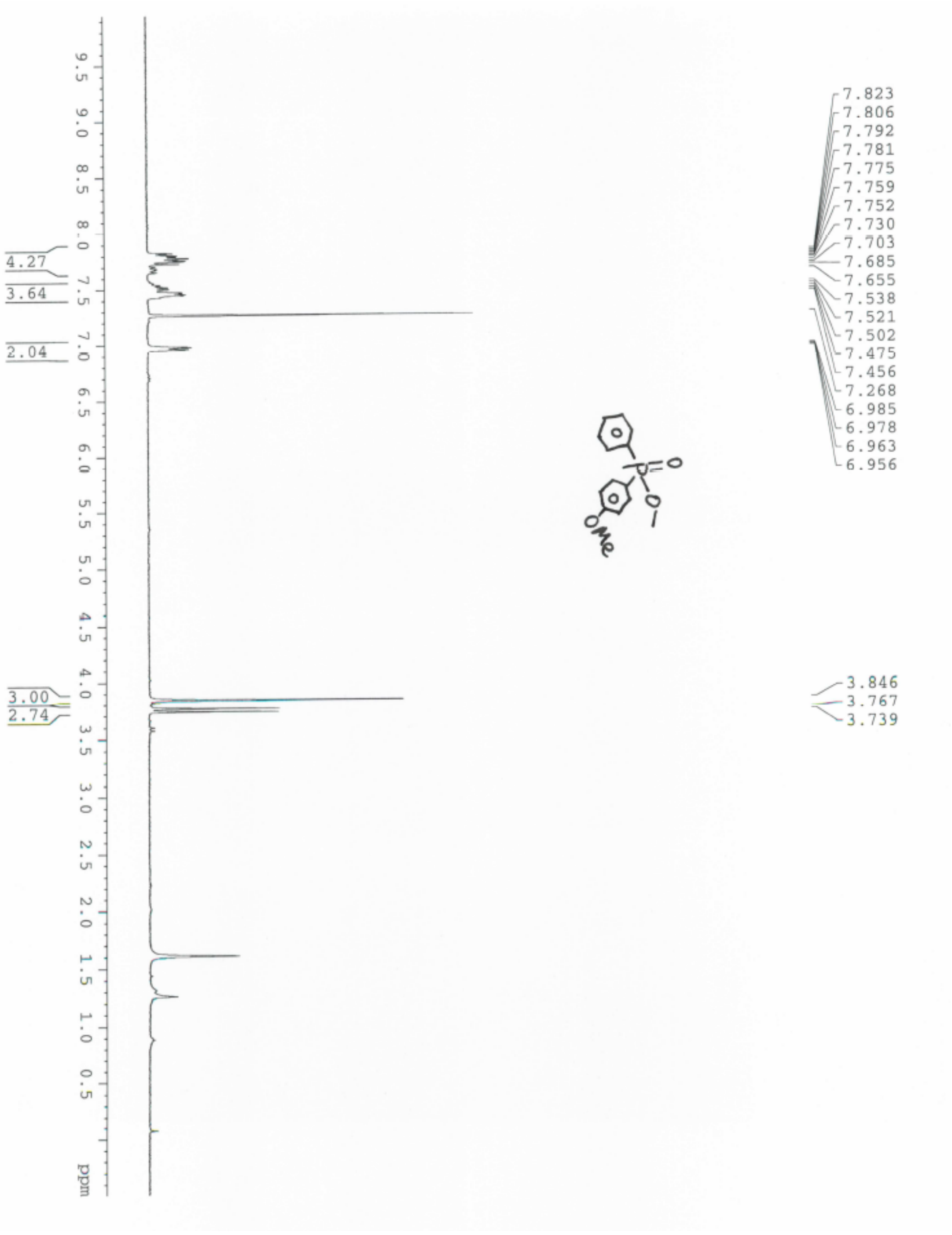
7.823
7.806
7.792
7.781
7.775
7.759
7.752
7.730
7.703
7.685
7.655
7.538
7.521
7.502
7.475
7.456
7.268
6.985
6.978
6.963
6.956
3.846
3.767
3.739
4.27
3.64
2.04
3.00
2.74
OMe
ppm

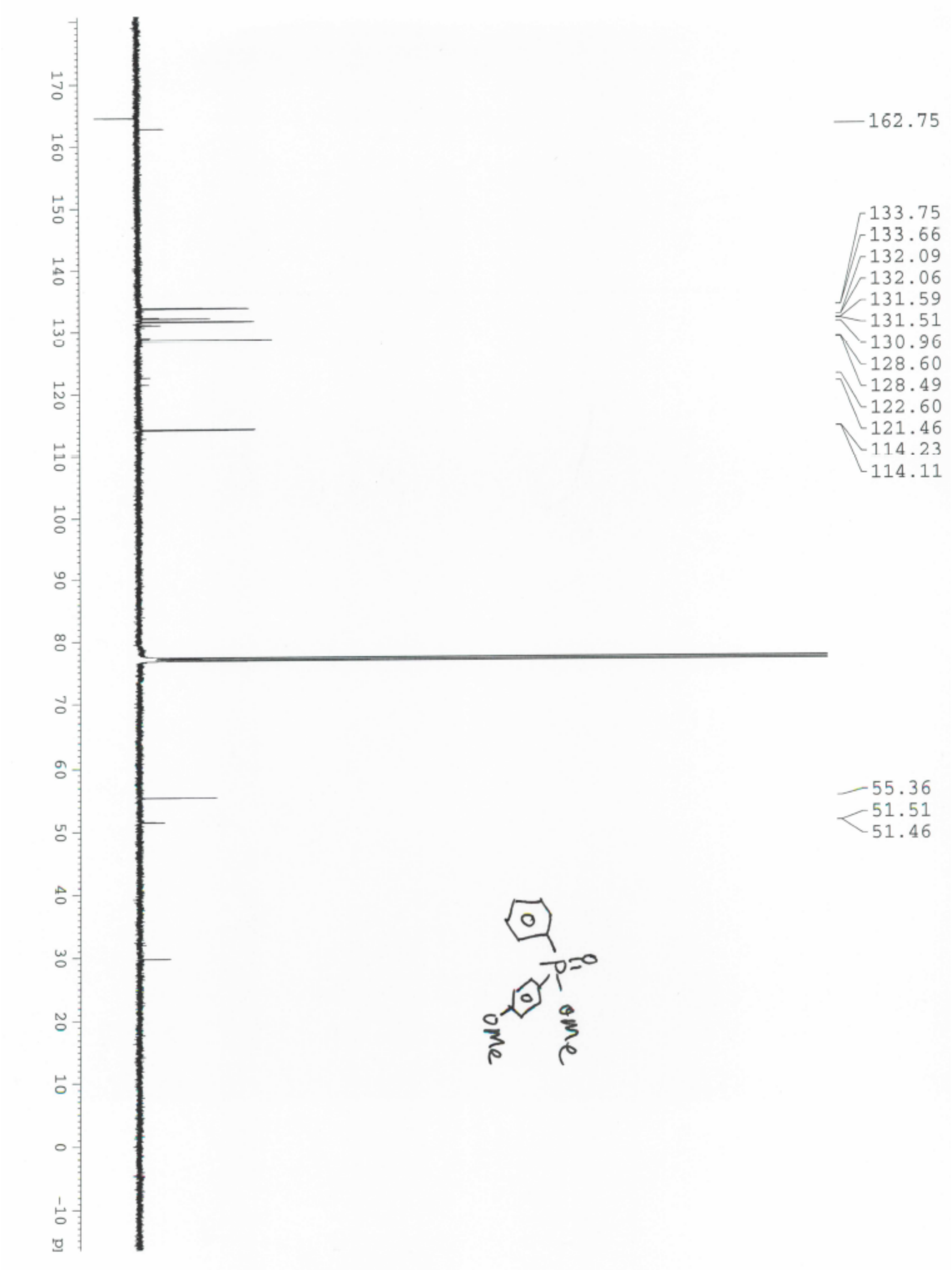
162.75
133.75
133.66
132.09
132.06
131.59
131.51
130.96
128.60
128.49
122.60
121.46
114.23
114.11
55.36
51.51
51.46

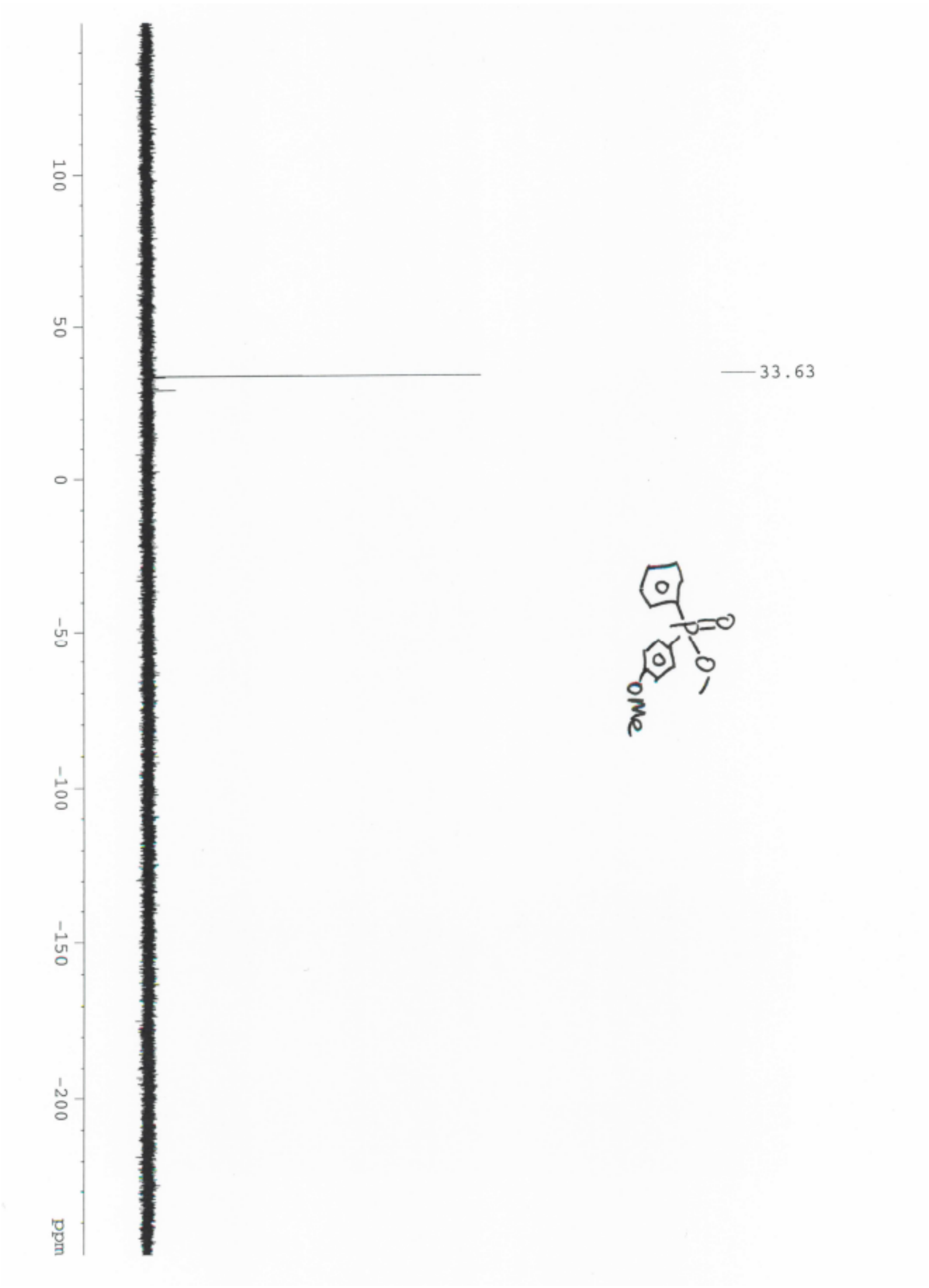

33.63
100
50
0
-50
-100
-150
-200
ppm
OMe

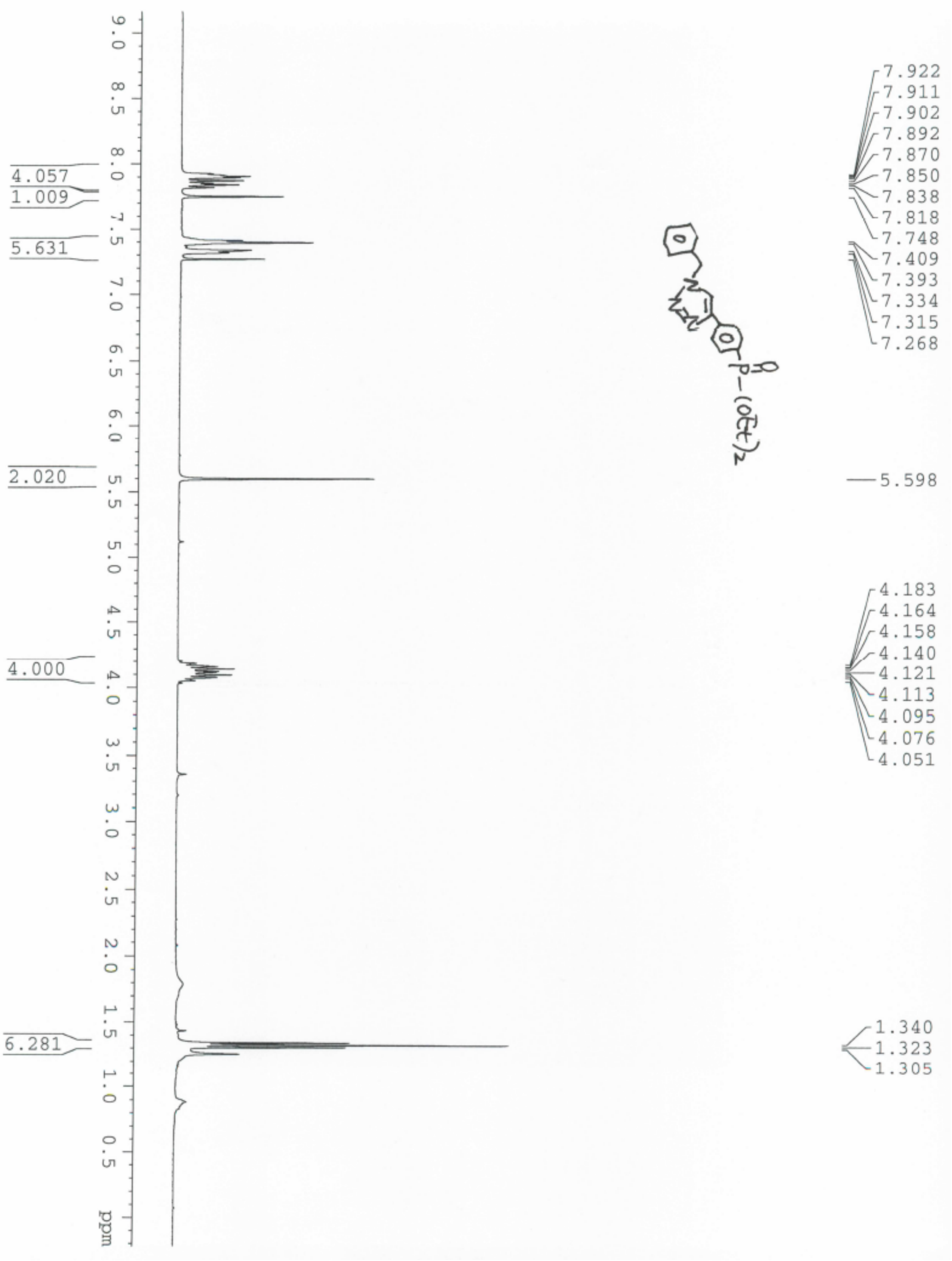
7.922
7.911
7.902
7.892
7.870
7.850
7.838
7.818
7.748
7.409
7.393
7.334
7.315
7.268
5.598
4.183
4.164
4.158
4.140
4.121
4.113
4.095
4.076
4.051
1.340
1.323
1.305
4.057
1.009
5.631
2.020
4.000
6.281
ppm

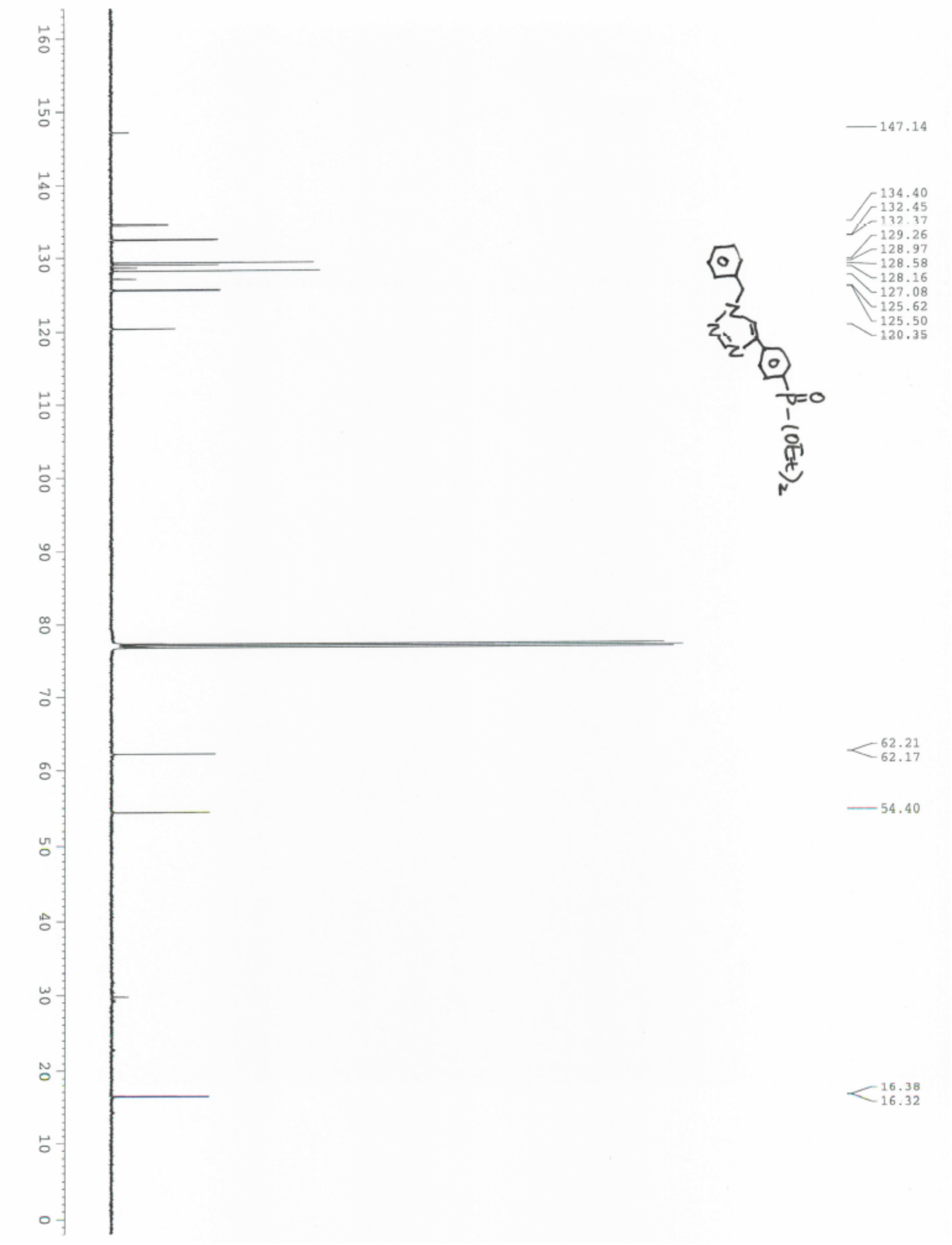
147.14
134.40
132.45
132.37
129.26
128.97
128.58
128.16
127.08
125.62
125.50
120.35
62.21
62.17
54.40
16.38
16.32

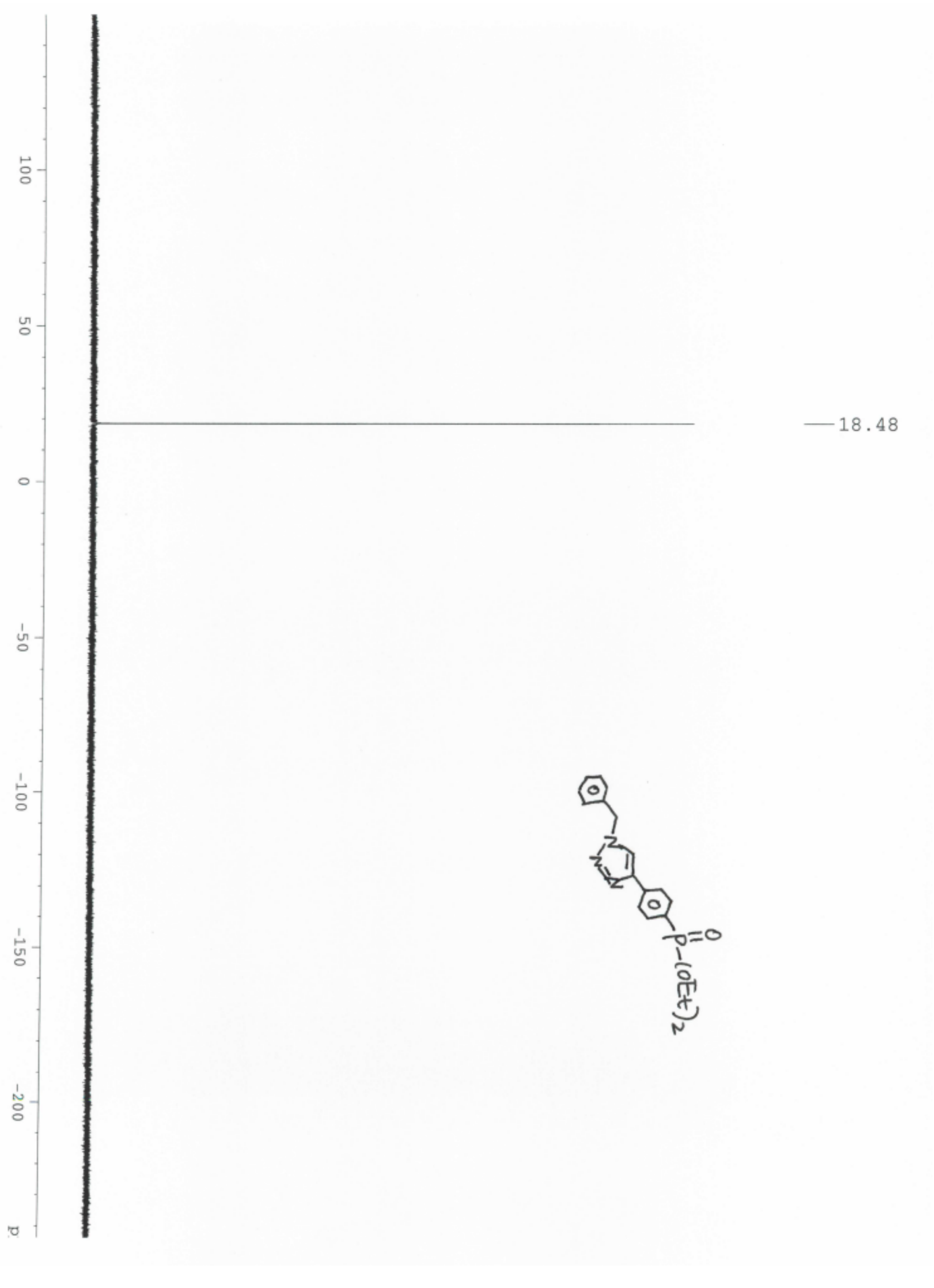
18.48
100
50
0
-50
-100
-150
-200
